# The IGF2BP3–FASN axis drives lipid metabolic reprogramming to promote brain colonization in non-small cell lung cancer

**DOI:** 10.1038/s41419-025-08006-z

**Published:** 2025-10-06

**Authors:** Jingwei Li, Shumin Ouyang, Ziyou Lin, Keren Peng, Jiayu Yan, Minyuan Lu, Wen Ding, Jianshan Mo, Yingxue Su, Libin Wang, Peibin Yue, Jin-Jian Lu, Xiangchao Yao, Yandong Wang, Xiaolei Zhang

**Affiliations:** 1 National-Local Joint Engineering Laboratory of Druggability and New Drug Evaluation, Guangdong Key Laboratory of Chiral Molecule and Drug Discovery, School of Pharmaceutical Sciences, Sun Yat-sen University, Guangzhou, 510006 China; 2 State Key Laboratory of Oncology in South China, Guangdong Provincial Clinical Research for Cancer, Sun Yat-sen University Cancer Center, Guangzhou, 510060 China; 3 State Key Laboratory of Ophthalmology, Zhongshan Ophthalmic Center, Sun Yat-sen University, Guangzhou, 510060 China; 4 Huazhong University of Science and Technology Union Shenzhen Hospital, Shenzhen, 518052 China; 5 Department of Medicine, Division of Hematology-Oncology, and Samuel Oschin Comprehensive Cancer Institute, Cedars-Sinai Medical Center, Los Angeles, CA 90048 USA; 6 State Key Laboratory of Quality Research in Chinese Medicine, Institute of Chinese Medical Sciences, University of Macau, Macao, China

**Keywords:** Non-small-cell lung cancer, Non-small-cell lung cancer

## Abstract

Brain metastases represent a significant cause of morbidity and mortality in non-small cell lung cancer (NSCLC), with limited therapeutic options. The unique brain microenvironment, characterized by low lipid availability, may drive NSCLC cells to adapt through lipid metabolic reprogramming. In this study, we identify a novel mechanism by which IGF2BP3-driven lipid metabolism promotes the brain colonization of NSCLC cells through the IGF2BP3-FASN axis. Elevated IGF2BP3 expression in NSCLC brain metastases correlates with poor prognosis and promotes cancer cell migration, invasion, and brain colonization by activating the lipogenesis pathway. We further identified that FASN was a downstream target of IGF2BP3 in NSCLC cells. Mechanistically, IGF2BP3 binds to FASN mRNA, enhancing its stability through RNA-binding activity. FASN is essential for neutral lipid accumulation and brain colonization, as demonstrated in vitro and in vivo. Our findings highlight the critical role of IGF2BP3 in lipid metabolism and propose that targeting IGF2BP3 may provide a promising therapeutic strategy for NSCLC brain colonization.

## Introduction

Lung cancer is the leading cause of cancer-related mortality worldwide, accounting for 18% of cancer deaths [1]. Non-small cell lung cancer (NSCLC) constitutes 80–85% of all lung cancer cases [2] and is frequently associated with brain metastases, occurring in 10–20% of patients at diagnosis and up to 40% during disease progression [3]. Current treatment options, including surgery, stereotactic radiotherapy, whole-brain radiotherapy, and chemotherapy, have limited efficacy against brain metastatic lesions, resulting in poor prognoses [4, 5]. The colonization step is a crucial part of brain metastasis formation, which depends on the survival and proliferation abilities of NSCLC cells within the brain microenvironment. Thus, understanding the mechanisms of brain colonization of NSCLC cells is essential for developing effective therapeutic strategies.

Metabolic reprogramming is a hallmark of cancer progression and metastasis [6]. Adaptation to the brain’s unique nutrient-deprived microenvironment, largely due to the restrictive blood-brain barrier (BBB), requires metabolic adjustments for cancer cells to survive and grow [7–9]. The brain’s limited extracellular lipid availability necessitates de novo lipid synthesis, a process heavily reliant on fatty acid synthase (FASN) [10]. FASN catalyzes the synthesis of fatty acids from acetyl-CoA, malonyl-CoA, and NADPH [11]. Elevated FASN expression has been observed in various cancers, including lung, breast, colorectal, and cholangiocarcinoma [12–16]. Xu and colleagues observed that FASN were significantly enriched in cholangiocarcinoma and FASN inhibitor significantly reversed the immunosuppressive microenvironment and enhanced anti-PD-1 efficacy in mouse model [13]. Recently, it has been reported that inhibition of FASN effectively reduce the brain metastasis burden in human epidermal growth factor receptor 2-positive breast cancer [17]. However, its role in NSCLC brain microenvironment adaption remains unclear.

RNA-binding proteins (RBPs) regulate post-transcriptional processes [18] and contribute to cancer metabolism and metastasis [19, 20]. Insulin-like growth factor 2 mRNA-binding protein 3 (IGF2BP3) is a member of RBPs consisting of two N-terminal RNA recognition binding regions (RRM) and four C-terminal heterologous ribonucleoprotein KH domains [21, 22]. IGF2BP3 is associated to tumor growth and migration in many kinds of cancer and related to poor prognosis [22–29]. Meanwhile, IGF2BP3 has been reported to be involved in tumor metastasis progression in gastric cancer, bladder cancer, cervical cancer and hepatocellular cancer [30–33]. Importantly, recent studies suggested that IGF2BP3 may be associated with the progression and prognosis of brain metastases in breast cancer and NSCLC [34, 35]. Nevertheless, the specific mechanisms of IGF2BP3 regulating brain colonization remain elusive.

In this study, we demonstrated that IGF2BP3-mediated lipid metabolism played a critical role in NSCLC brain colonization. IGF2BP3 is upregulated in NSCLC brain metastases and correlated with poor prognosis. Inhibition of IGF2BP3 triggers a remarkable reprogramming of cellular metabolites, especially those involved in the lipid metabolism, resulting in the reduction of total triglycerides, cholesterol, lipid droplets and neutral lipid level of NSCLC cells. Furthermore, we revealed that targeting IGF2BP3 significantly suppressed NSCLC cell growth within the brain microenvironment and neutral lipid accumulation in mouse model and this effect was dependent on the FASN, a key downstream target of IGF2BP3 in lipogenesis. These findings establish the IGF2BP3–FASN axis as a critical regulator of NSCLC lipid metabolism and brain colonization and highlight IGF2BP3 as a potential therapeutic target in NSCLC brain metastasis.

## Results

### IGF2BP3 is upregulated in NSCLC brain metastasis and associated with the poor prognosis

**Fig. 1:**
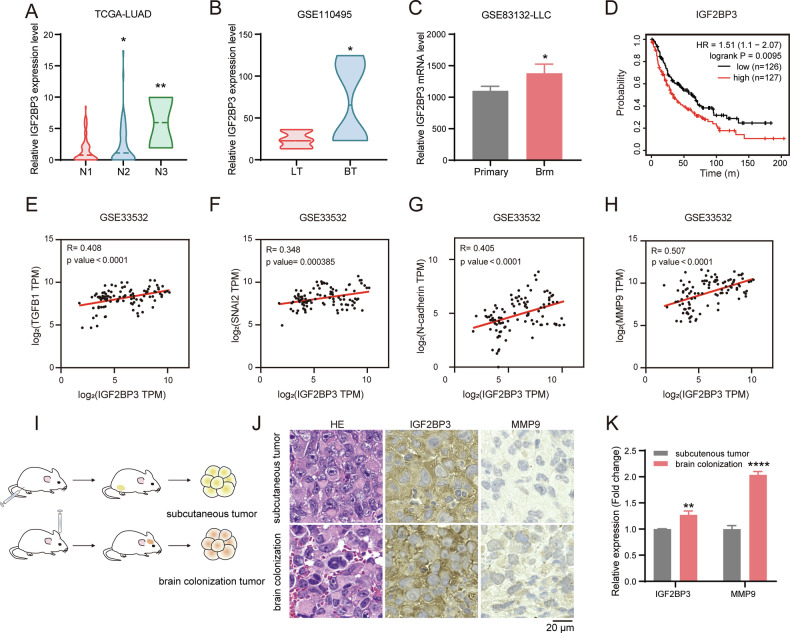
IGF2BP3 is upregulated in NSCLC brain metastasis and associated with poor prognosis. **A** IGF2BP3 expression was elevated in N2 (*n* = 74) and N3 (*n* = 2) stage metastatic tumor compared with N1 stage metastatic tumor (*n* = 95) in lung adenocarcinoma (LUAD) patients with lymph node metastasis according to TCGA database. Data are presented as median value. **B** IGF2BP3 expression levels were elevated in brain metastatic tumors (BT) (*n* = 6) compared with primary lung tumors (LT) (*n* = 8) in early passage cell lines derived from primary patient samples of lung-to-brain metastases according to GSE110495 dataset. Data are presented as median value. **C** IGF2BP3 expression levels were elevated in LLC-derived brain metastatic cells (Brm) compared with primary cells according to GSE83132 dataset. Data are presented as mean ± SD; *n* = 3. **D** Overall survival analysis of LUAD patients at the N1 stage based on TCGA project was calculated with the log-rank test and stratified by high or low IGF2BP3 expression (high: top 50%, *n* = 127; low: bottom 50%, *n* = 126). **E**–**H** Pearson correlation analysis between IGF2BP3 expression and key EMT markers (TGFB1, SNAI2, N-cadherin, and MMP9) in LUAD patients according to GSE33532 dataset (*n* = 100). **I** Schematic of the subcutaneous tumor and stereotactic brain injection tumor model. **J** Hematoxylin and eosin (H&E) staining and immunohistochemistry (IHC) analysis of IGF2BP3 and MMP9 in representative subcutaneous tumor and xenograft tumor in the brain from mice. Scale bar = 20 μm. **K** Quantification of IGF2BP3 and MMP9 expression levels from IHC analysis in **J** using Image Pro Plus. Data are presented as mean ± SD; *n* = 3. *P < 0.05; **P < 0.01; ****P < 0.0001 (two-tailed *t*-test).

To investigate the role of IGF2BP3 in NSCLC brain metastasis, we analyzed multiple NSCLC cohorts with metastasis using data from the TCGA and GEO databases. IGF2BP3 expression was significantly upregulated in advanced metastatic stages in the TCGA cohort (Fig. 1A). Consistent with this, IGF2BP3 levels were higher in brain metastases compared to primary lung tumors in NSCLC from the GEO cohorts (GSE110495 and GSE83132) (Fig. 1B, C). Kaplan-Meier survival analysis further revealed that elevated IGF2BP3 expression correlated with poor prognosis in NSCLC patients with metastasis (Fig. 1D). Pearson correlation analysis demonstrated significant positive associations between IGF2BP3 and key epithelial-mesenchymal transition (EMT) markers, including TGFB1, SNAI2, N-cadherin, and MMP9, in human NSCLC tumors (Fig. 1E–H). To validate these findings in vivo, we established subcutaneous tumor model and a stereotactic brain injection model in mice and assessed IGF2BP3 protein expression (Fig. 1I). Immunohistochemistry (IHC) staining revealed significantly higher IGF2BP3 levels in brain colonization tumor compared to subcutaneous tumors (Fig. 1J, K). Concurrently, MMP9 protein expression was also elevated in brain colonization tumor (Fig. 1J, K). Collectively, these findings suggest a potential role for IGF2BP3 in regulating NSCLC brain metastasis.

### IGF2BP3 promotes the growth of NSCLC cells in the brain

**Fig. 2:**
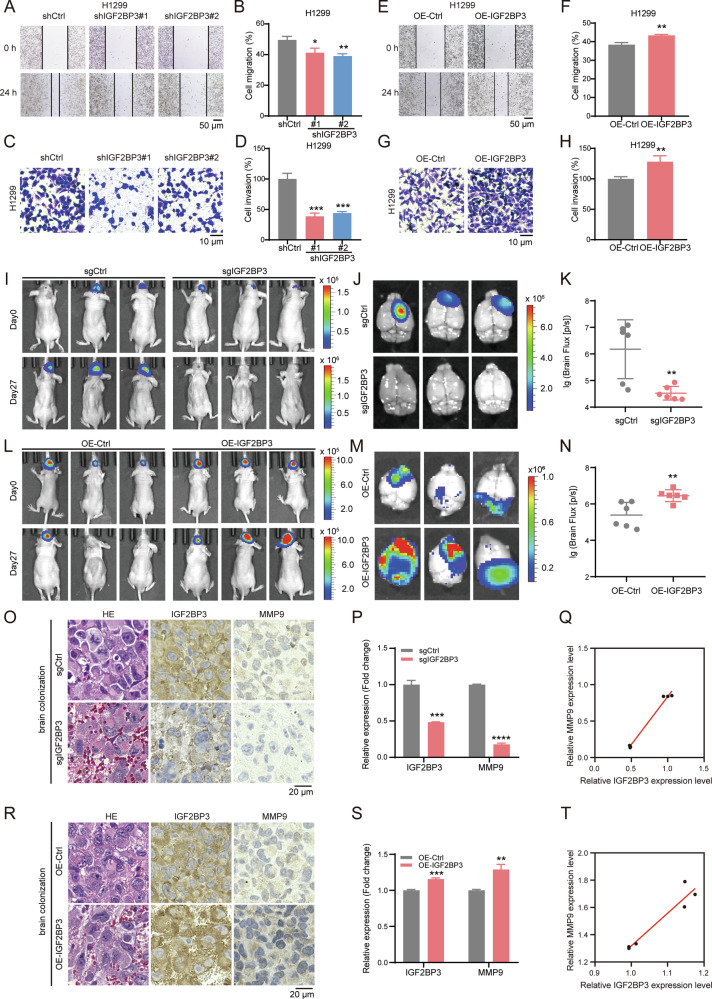
IGF2BP3 promotes the growth of NSCLC cells in the brain. **A** Wound healing assay showing cell migration ability in H1299 cells treated with shRNAs targeting IGF2BP3 or control (shCtrl) after 24 h. Scale bars = 50 μm. **B** Quantification of cell migration in IGF2BP3-knockdown H1299 cells from (**A**) using ImageJ. Data are presented as mean ± SD; *n* = 3. **C** Transwell invasion assay showing cell invasion ability in H1299 cells treated with shRNAs targeting IGF2BP3 or control after 24 h. Scale bars = 10 μm. **D** Quantification of cell invasion in IGF2BP3-knockdown H1299 cells from (**C**) using ImageJ. Data are presented as mean ± SD; *n* = 3. **E** Wound healing assay showing cell migration ability in H1299 cells overexpressing IGF2BP3 (OE-IGF2BP3) or control after 24 h. Scale bars = 50 μm. **F** Quantification of cell migration in IGF2BP3-overexpressing H1299 cells from (**E**) using ImageJ. Data are presented as mean ± SD; *n* = 3. **G** Transwell invasion assay showing cell invasion ability in H1299 cells overexpressing IGF2BP3 or control after 24 h. Scale bars = 10= μm. **H** Quantification of cell invasion in IGF2BP3-overexpressing H1299 cells from (**G**) using ImageJ. Data are presented as mean ± SD; *n* = 3. **I**–**K**. H1299-luc cells (5 × 10^5^) with IGF2BP3 knockout (sgIGF2BP3) or control (sgCtrl) were injected into BALB/c-nu/nu mice via stereotactic brain injection (*n* = 6 per group). Representative bioluminescent images of NSCLC xenograft tumor in the brain captured by the Neurostar IVIS preclinical in vivo imaging system on day 27 are shown in (**I**, **J**). Quantification of bioluminescence in the brain on day 27 is shown in (**K**). Data are presented as mean ± SD; *n* = 6. **L**–**N** H1299-luc cells (5 × 10^5^) overexpressing IGF2BP3 (OE-IGF2BP3) or control were injected into BALB/c-nu/nu mice via stereotactic brain injection (*n* = 6 per group). Representative bioluminescent images of NSCLC xenograft tumor in the brain on day 27 are shown in (**L**, **M**). Quantification of bioluminescence in the brain on day 27 is shown in (**N**). Data are presented as mean ± SD; *n* = 6. **O** H&E staining and IHC analysis of IGF2BP3 and MMP9 in representative brain tumors from IGF2BP3-knockout and control mice. Scale bar = 20 μm. **P** Quantification of IGF2BP3 and MMP9 expression levels from IHC analysis in (**O**) using Image Pro Plus. Data are presented as mean ± SD; *n* = 3. **Q** IHC staining depicted the correlation between IGF2BP3 and MMP9 expression in brain tumors from IGF2BP3-knockout and control mice (*n* = 3). **R** H&E staining and IHC analysis of IGF2BP3 and MMP9 in representative brain tumors from IGF2BP3-overexpressing and control mice. Scale bar = 20 μm. **S** Quantification of IGF2BP3 and MMP9 expression levels from IHC analysis in (**R**) using Image Pro Plus. Data are presented as mean ± SD; *n* = 3. **T** IHC staining depicted the correlation between IGF2BP3 and MMP9 expression in brain tumors from IGF2BP3-overexpressing and control mice (*n* = 3). *P < 0.05; **P < 0.01; ***P < 0.001; ****P < 0.0001 (two-tailed *t*-test).

To determine the functional role of IGF2BP3 in NSCLC invasiveness, we generated IGF2BP3-knockdown (shIGF2BP3) and IGF2BP3-overexpressing (OE-IGF2BP3) H1299 and H157 cell lines. Knockdown and overexpression were confirmed at the mRNA and protein levels by RT-qPCR and immunoblotting, respectively (Figs. S1A–D and S3A–D). Functional assays demonstrated that IGF2BP3 knockdown reduced cell proliferation (Fig. S1E–H) and colony formation (Fig. S1I–K) while increasing apoptosis (Fig. S1M–P). Importantly, IGF2BP3 knockdown significantly inhibited cell migration (Figs. 2A, B and S2A, B) and invasion (Fig. 2C, D and S2C, D). Conversely, IGF2BP3 overexpression enhanced proliferation (Fig. S3E–H), colony formation (Fig. S3I–K), migration (Fig. 2E, F and S2E, F) and invasion (Fig. 2G, H and S2G, H), while reducing apoptosis (Fig. S3L–O) in H1299 and H157 cell lines.

Furthermore, we measured the ability of NSCLC cells to colonize the brain, and established a mouse stereotactic brain injection model using luciferase-tagged H1299 cells (Fig. S2I). Firstly, we constructed stable IGF2BP3-knockout and IGF2BP3 overexpression H1299 cell lines with luciferase (sgIGF2BP3-luc and OE-IGF2BP3-luc, respectively). The protein expression of IGF2BP3 was verified by immunoblotting (Figs. S1L and S3P). H1299 cells were injected into the cortex of 4-week-old nude mice and the body weight was measured every three days (Fig. S2J, K). The tumors were imaged in vivo by IVIS system every nine days and plotted a growth curve. Next, the mice brains were harvested and imaged on day 27. IGF2BP3 knockout significantly reduced the growth of NSCLC cells in the brain (Figs. 2I–K, S2L), whereas IGF2BP3 overexpression exacerbated tumor burden (Fig. 2L–N and S2M). IHC staining confirmed IGF2BP3 expression changes in brain colonization tumor with MMP9 expression patterns aligning with IGF2BP3 levels (Fig. 2O–T). These results indicate that IGF2BP3 promotes the growth of NSCLC cells in the brain, highlighting its potential as a therapeutic target.

### IGF2BP3 mediates lipid metabolism in NSCLC

**Fig. 3:**
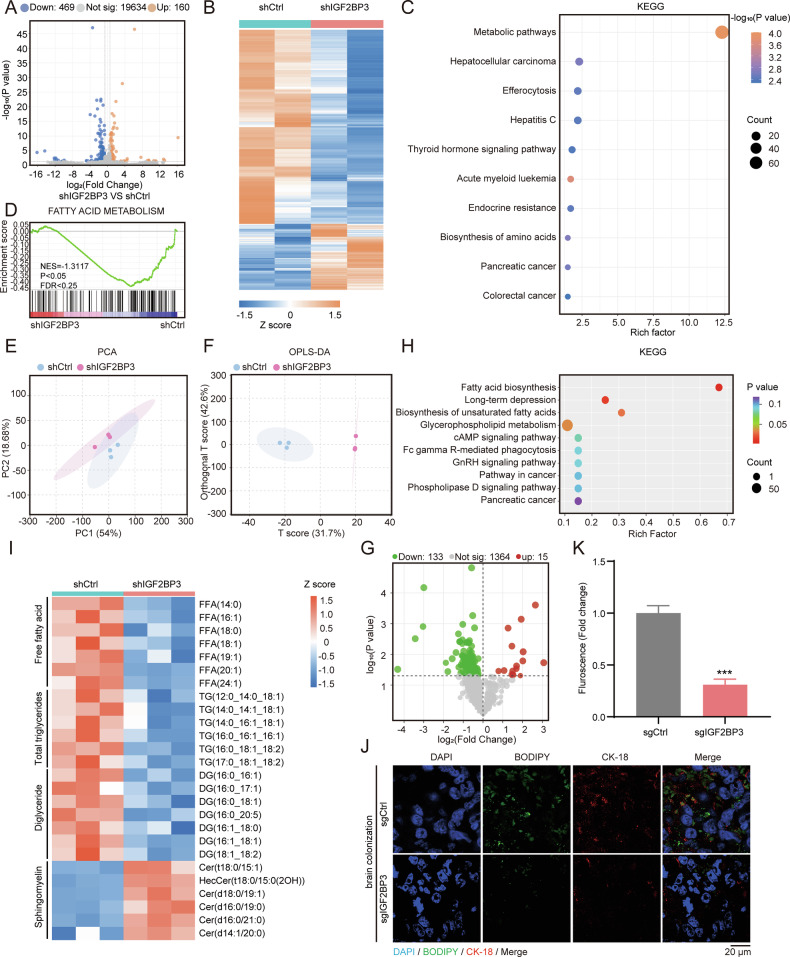
IGF2BP3 mediates lipid metabolism in NSCLC. **A** Volcano plot of differentially expressed genes (fold change > 1.5, *p* < 0.05) in H1299 cells stably expressing shRNAs targeting IGF2BP3 (shIGF2BP3) or scramble control (shCtrl). **B** Heatmap of differentially expressed genes (fold change > 1.5, *p* < 0.05) between IGF2BP3-knockdown and control H1299 cells measured by *z*-score. The color means the *z* score of cluster analysis (**C**) KEGG enrichment analysis of differentially expressed genes (fold change > 1.5, *p* < 0.05) identified by RNA-seq in IGF2BP3-knockdown H1299 cells. The color means −log10 (*p* value) and the dot size means gene count of different pathways. **D** Gene Set Enrichment Analysis (GSEA) showing decreased fatty acid metabolism in IGF2BP3-knockdown cells. **E**, **F** Principal component analysis (PCA) and orthogonal partial least squares-discriminant analysis (OPLS-DA) of lipidomics data in IGF2BP3-knockdown H1299 cells. **G** Volcano plot of differential metabolites (fold change > 1.5, *p* < 0.05) in IGF2BP3-knockdown H1299 cells. **H** KEGG enrichment analysis of differential metabolites (fold change > 1.5, *p* < 0.05) identified by lipidomics in IGF2BP3-knockdown H1299 cells. The color means *p* value and dot size means gene count of pathways. **I** Heatmap of differential metabolites (fold change > 1.5, *p* < 0.05) between IGF2BP3-knockdown and control H1299 cells measured by *z*-score. The color means the *z* score of cluster analysis. **J**, **K** Neutral lipid levels detected by BODIPY 493/503 staining in IGF2BP3-knockout brain tumors examined by microscope. Representative fluorescent images (**J**) and quantification (**K**) are shown. Data are presented as mean ± SD; *n* = 3. ***P < 0.001 (two-tailed *t*-test).

To elucidate the mechanism by which IGF2BP3 promotes the growth of NSCLC cells in the brain, we performed transcriptome sequencing on IGF2BP3-knockdown (shIGF2BP3) and control (shCtrl) H1299 cells. We identified 160 upregulated and 469 downregulated genes (fold change ≥1.5, *p* ≤ 0.05) (Fig. 3A, B). Gene enrichment analysis revealed significant alterations in metabolic pathways (Fig. 3C). GSEA analysis further linked IGF2BP3 to fatty acid metabolism (Fig. 3D).

To further examine the changes in metabolites related to lipid metabolism, we next performed lipomics analysis via LC-MS and carried out multivariate statistical analysis by principal component analysis (PCA) and orthogonal PLS-DA (OPLS-DA) (Fig. 3E, F). Lipomics analysis indicated that 15 upregulated and 133 downregulated metabolites in shIGF2BP3 H1299 cell lines (Fig. 3G). KEGG enrichment analysis indicated dysregulation in fatty acid biosynthesis and unsaturated fatty acid biosynthesis pathways (Fig. 3H). Heatmaps highlighted key metabolite changes, with IGF2BP3 knockdown reducing fatty acid, triglyceride, and diglyceride levels (Fig. 3I). Immunofluorescence staining of xenograft tumor in the brain confirmed decreased neutral lipid accumulation in IGF2BP3-knockout tumors (Fig. 3J, K).

**Fig. 4:**
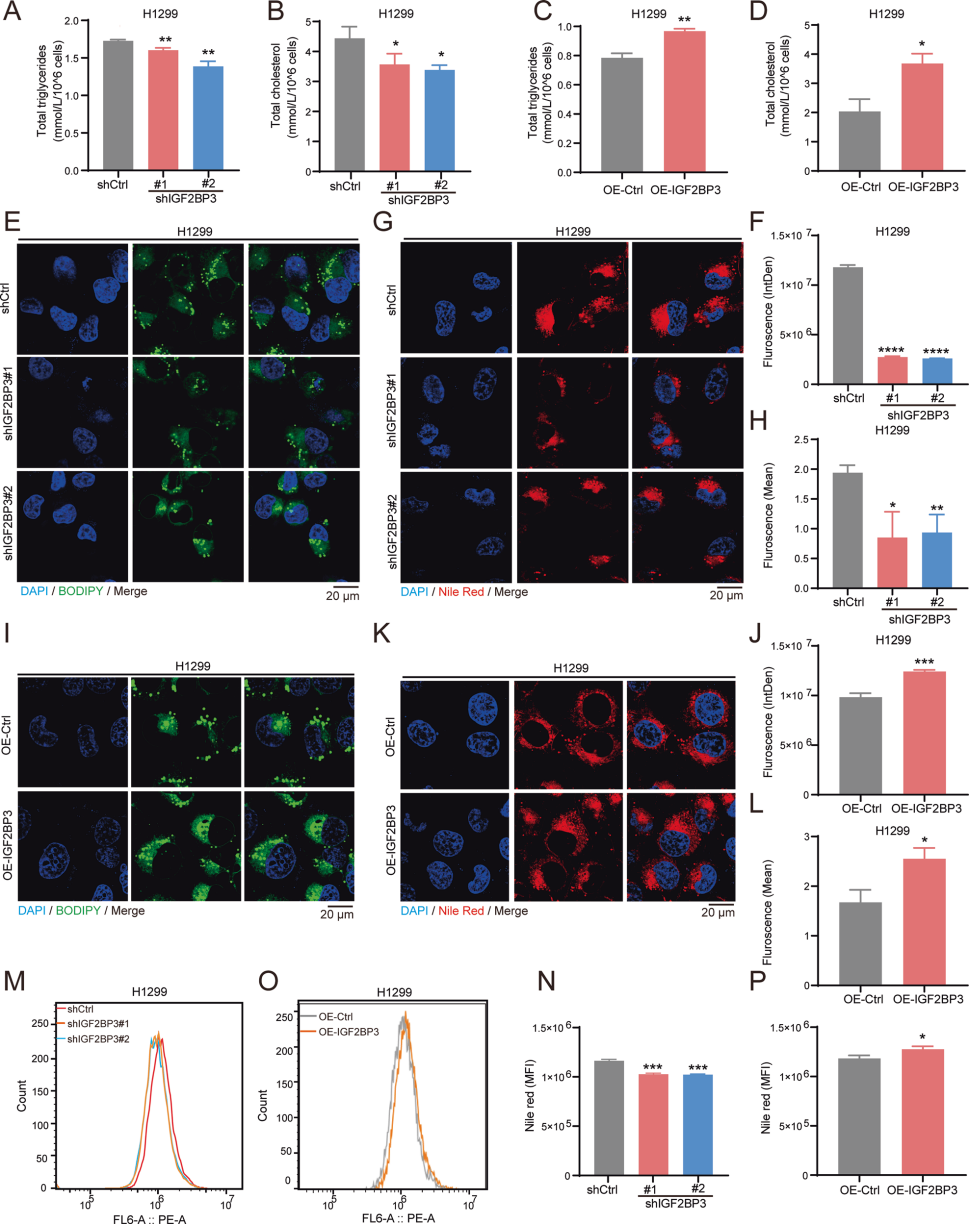
IGF2BP3 regulates lipid metabolism in NSCLC cells. **A** Total triglycerides assay kit showing total triglyceride levels were decreased in IGF2BP3-knockdown H1299 cells compared with control cells. Data are presented as mean ± SD; *n* = 3. **B** Total cholesterol assay kit showing total cholesterol levels were decreased in IGF2BP3-knockdown H1299 cells compared with control cells. Data are presented as mean ± SD; *n* = 3. **C** Total triglycerides assay kit showing total triglyceride levels were elevated in IGF2BP3-overexpressing H1299 cells compared with control cells. Data are presented as mean ± SD; *n* = 3. **D** Total cholesterol assay kit showing total cholesterol levels were elevated in IGF2BP3-overexpressing H1299 cells compared with control cells. Data are presented as mean ± SD; *n* = 3. **E**, **F** Neutral lipid levels detected by BODIPY 493/503 staining in IGF2BP3-knockdown H1299 cells. Representative fluorescent images (**E**) and quantification (**F**) are shown. Data are presented as mean ± SD; *n* = 3. **G**, **H** Neutral lipid levels measured by Nile Red staining in IGF2BP3-knockdown H1299 cells. Representative fluorescent images (**G**) and quantification (**H**) are shown. Data are presented as mean ± SD; *n* = 3. **I**, **J** Neutral lipid levels detected by BODIPY 493/503 staining in IGF2BP3-overexpressing H1299 cells. Representative fluorescent images (**I**) and quantification (**J**) are shown. Data are presented as mean ± SD; *n* = 3. **K**, **L** Neutral lipid levels measured by Nile Red staining in IGF2BP3-overexpressing H1299 cells. Representative fluorescent images (**K**) and quantification (**L**) are shown. Data are presented as mean ± SD; *n* = 3. **M**, **N** Neutral lipid levels analyzed by Nile Red staining using flow cytometry in IGF2BP3-knockdown H1299 cells. Representative images (**M**) and quantification (**N**) are shown. Data are presented as mean ± SD; *n* = 3. **O**, **P** Neutral lipid levels analyzed by Nile Red staining using flow cytometry in IGF2BP3-overexpressing H1299 cells. Representative images (**O**) and quantification (**P**) are shown. Data are presented as mean ± SD; *n* = 3. *P < 0.05; **P < 0.01; ***P < 0.001; ****P < 0.0001 (two-tailed *t*-test).

Functional assays further supported IGF2BP3’s role in lipid metabolism. IGF2BP3 knockdown reduced total triglycerides and cholesterol levels (Figs. 4A, B and S4A-B). Then, we used the BODIPY 493/503 probe to detect the neutral lipid levels, via fluorescent microscope. The result confirmed that IGF2BP3 knockdown significantly inhibits the neutral lipid levels (Figs. 4E, F and S4E, F). Furthermore, we found that IGF2BP3 knockdown caused a decrease of lipid droplets (Figs. 4G, H and S4G-H), using Nile red which is a dye commonly used to measure lipid droplets. Flow cytometry further confirmed reduced Nile red staining in IGF2BP3-knockdown cells (Figs. 4M, N and S4M, N). Conversely, IGF2BP3 overexpression increased lipid accumulation, including triglycerides (Figs. 4C and S4C), cholesterol (Figs. 4D and S4D), neutral lipids (Figs. 4I, J and S4I, J), and lipid droplets (Figs. 4K, L, O–P, S4K, L and S4O, P). These findings establish IGF2BP3 as a critical regulator of lipid metabolism in NSCLC cells.

### IGF2BP3 stabilizes FASN mRNA to regulate lipid metabolism in NSCLC cells

**Fig. 5:**
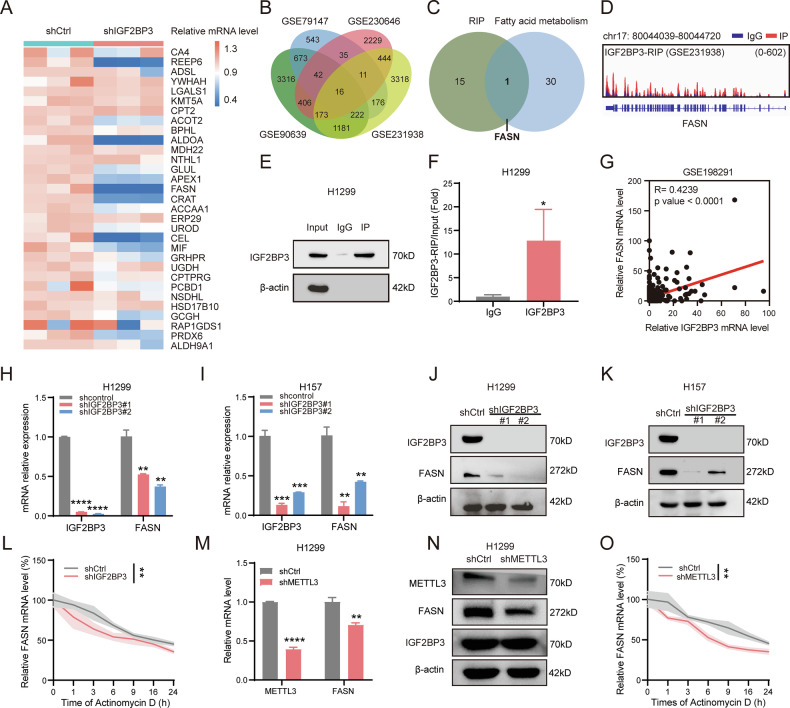
IGF2BP3 stabilizes FASN mRNA to regulate lipid metabolism in NSCLC cells. **A** RT-qPCR analysis of fatty acid metabolism-related genes with cDNA microarrays in IGF2BP3-knockdown H1299 cells (*n* = 3). The color means the relative mRNA expression level of indicated genes. **B** Overlapping analysis of IGF2BP3-regulated genes detected by RIP-seq in GSE90639, GSE79147, GSE230646, and GSE231938 (fold change > 1.5, *p* < 0.05). **C** Overlapping analysis of genes from (**A**) and (**B**). **D** RIP-seq data showing potential binding of FASN mRNA to IGF2BP3 according to GSE231938 dataset. **E** RIP assay in IGF2BP3-overexpressing H1299 cells were verified by Western blotting. **F** RIP-qPCR analysis of FASN mRNA enrichment in IGF2BP3-overexpressing H1299 cells. Data are presented as mean ± SD; *n* = 3. **G** Pearson correlation analysis of IGF2BP3 and FASN expression in metastasis tumor tissue of LUAD patients according to GSE198291 dataset (*n* = 312). **H**, **I** FASN mRNA expression evaluated by RT-qPCR in IGF2BP3-knockdown H1299 and H157 cells. Data are presented as mean ± SD; *n* = 3. **J**, **K** FASN protein expression evaluated by Western blotting in IGF2BP3-knockdown H1299 and H157 cells. **L** FASN mRNA half-life assessed by RT-qPCR in IGF2BP3-knockdown H1299 cells treated with 20 μg/mL actinomycin D for 0, 1, 3, 6, 9, 16, and 24 hours. Data are presented as mean ± SD; *n* = 3. **M** FASN mRNA expression evaluated by RT-qPCR in shCtrl and shMETTL3 H1299 cells. Data are presented as mean ± SD; *n* = 3. **N** FASN and IGF2BP3 protein levels evaluated by Western blotting in shCtrl and shMETTL3 H1299 cells. **O** FASN mRNA half-life assessed by RT-qPCR in METTL3-knockdown H1299 cells treated with 20 μg/mL actinomycin D for 0, 1, 3, 6, 9, 16, and 24 h. Data are presented as mean ± SD; *n* = 3. *P < 0.05; **P < 0.01; ***P < 0.001; ****P < 0.0001 (two-tailed *t*-test).

Given the role of IGF2BP3 in lipid production regulation, we sought to identify its potential binding targets in lipid metabolism. We carried out cDNA microarrays analysis, which demonstrated the expression of fatty acid metabolism-related genes in H1299 cells after IGF2BP3 knockdown. We found that the key genes of fatty acid metabolism including REEP6, ALDOA, FASN, CRAT and CEL were markedly down-regulated after IGF2BP3 knockdown(Fig. 5A). Analysis of four independent IGF2BP3-RIP datasets from the GEO database identified 16 potential IGF2BP3-binding mRNAs (Fig. 5B). Overlapping these genes with fatty acid metabolism-related signatures highlighted FASN, a key enzyme in the endogenous lipogenesis pathway [36], as a potential target of IGF2BP3-mediated RNA binding (Fig. 5C).

To validate this interaction, we analyzed the IGF2BP3-RIP seq data (Fig. 5D) and performed RIP-qPCR and confirmed that IGF2BP3 directly binds to FASN mRNA in NSCLC cells (Fig. 5E, F). Analysis of the GSE198291 dataset further demonstrated a significant positive correlation between IGF2BP3 and FASN mRNA expression in metastasis tumor tissue of LUAD patients (Fig. 5G). RT-qPCR and immunoblotting analyses revealed that FASN expression was significantly downregulated upon IGF2BP3 knockdown in NSCLC cells (Fig. 5H–K). Additionally, the half-life of FASN mRNA was markedly reduced in IGF2BP3-knockdown H1299 cells, indicating that IGF2BP3 stabilizes FASN mRNA (Fig. 5L). Previous studies have shown that IGF2BP3 is an m6A reading protein [29, 37], we investigated whether IGF2BP3 regulates FASN translation in an m6A-dependent manner. We established m6A-deficient NSCLC cells by knocking down METTL3 [38], a crucial m6A methyltransferase, to measure the mRNA and protein levels of FASN. Our results revealed the down-regulation of FASN expression while IGF2BP3 remained unchanged (Fig. 5M, N). Consistent with this, the half-life of FASN mRNA was significantly reduced in METTL3-knockdown cells compared to controls (Fig. 5O). Furthermore, we detected the mRNA levels of FASN in stable IGF2BP3-konckdown and METTL3-overexpression NSCLC cells. Our results revealed that forced expression of METTL3 rescued the expression of FASN in IGF2BP3-deficient NSCLC cells (Fig. S5A). These findings suggest that FASN is a direct target of IGF2BP3 in regulating lipid metabolism in NSCLC cells.

As a downstream target of IGF2BP3, FASN may play a critical role in lipogenesis and metastasis. To assess this, we generated stable FASN-knockdown NSCLC cell lines and evaluated their functional phenotypes (Fig. S5B-E). Our results showed that FASN knockdown reduced cell proliferation (Fig. S5F-I), colony formation (Fig. S5J–L), cell invasion (Figs. S6A, B and S7A, B) and migration (Fig. S6C, D and S7C, D). The flow cytometry analysis demonstrated that FASN knockdown increased cell apoptosis (Fig. S5M–P). Subsequently, we also explored the role of FASN in lipogenesis in NSCLC cells. FASN knockdown decreased total triglycerides and cholesterol levels (Figs. S6E, F and S7E, F), reduced lipid droplet formation (Figs. S6G, H, S6K, L, S7G, H, S7K, L), and lowered neutral lipid levels (Figs. S6I, J and S7I, J). These results demonstrate that FASN is essential for lipid metabolism and invasiveness in NSCLC cells.

### The IGF2BP3-FASN axis mediates lipid metabolism to promote brain colonization in NSCLC

**Fig. 6:**
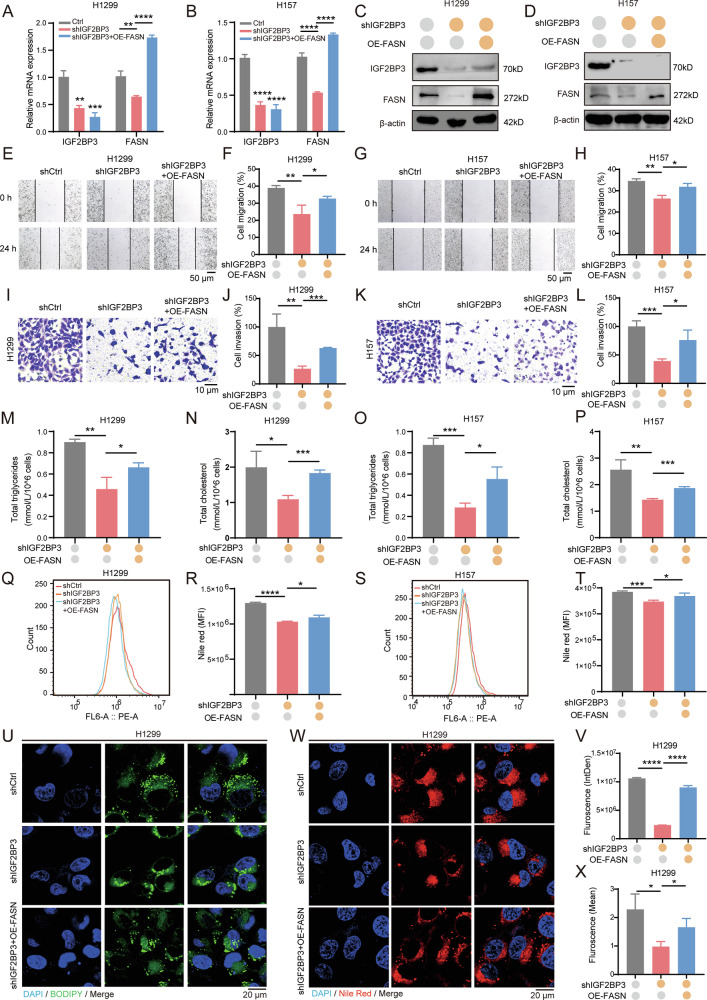
IGF2BP3-FASN axis regulates invasiveness and lipid metabolism in NSCLC cells. **A**, **B** IGF2BP3 and FASN mRNA expression evaluated by RT-qPCR in IGF2BP3-knockdown and FASN-overexpressing H1299 and H157 cells. Data are presented as mean ± SD; *n* = 3. **C**, **D** IGF2BP3 and FASN protein levels evaluated by Western blotting in IGF2BP3-knockdown and FASN-overexpressing H1299 and H157 cells. **E** Wound healing assay showing cell migration ability in H1299 cells treated with IGF2BP3 knockdown and FASN overexpression for 24 h. Scale bars = 50 μm. **F** Quantification of cell migration in IGF2BP3-knockdown and FASN-overexpressing H1299 cells from (**E**) using ImageJ. Data are presented as mean ± SD; *n* = 3. **G** Wound healing assay showing cell migration ability in H157 cells treated with IGF2BP3 knockdown and FASN overexpression for 24 h. Scale bars = 50 μm. **H** Quantification of cell migration in IGF2BP3-knockdown and FASN-overexpressing H157 cells from (**G**) using ImageJ. Data are presented as mean ± SD; *n* = 3. **I** Transwell invasion assay showing invasion ability in H1299 cells treated with IGF2BP3 knockdown and FASN overexpression for 24 h. Scale bars = 10 μm. **J** Quantification of cell invasion in IGF2BP3-knockdown H1299 cells from (**I**) using ImageJ. Data are presented as mean ± SD; *n* = 3. **K** Transwell invasion assay showing invasion ability in H157 cells treated with IGF2BP3 knockdown and FASN overexpression for 24 h. Scale bars = 10 μm. **L** Quantification of cell invasion in IGF2BP3-knockdown H157 cells from (**K**) using ImageJ. Data are presented as mean ± SD; *n* = 3. **M** Total triglycerides assay kit showing total triglyceride levels were rescued in IGF2BP3-knockdown and FASN-overexpressing H1299 cells compared with IGF2BP3-knockdown cells. Data are presented as mean ± SD; *n* = 3. **N** Total cholesterol assay kit showing total cholesterol levels were rescued in IGF2BP3-knockdown and FASN-overexpressing H1299 cells compared with IGF2BP3-knockdown cells. Data are presented as mean ± SD; *n* = 3. **O** Total triglycerides assay kit showing total triglyceride levels were rescued in IGF2BP3-knockdown and FASN-overexpressing H157 cells compared with IGF2BP3-knockdown cells. Data are presented as mean ± SD; *n* = 3. **P** Total cholesterol assay kit showing total cholesterol levels were rescued in IGF2BP3-knockdown and FASN-overexpressing H157 cells compared with IGF2BP3-knockdown cells. Data are presented as mean ± SD; *n* = 3. **Q**–**T** Neutral lipid levels analyzed by flow cytometry in IGF2BP3-knockdown and FASN-overexpressing H1299 and H157 cells stained with Nile Red. Representative images (**Q**, **S**) and quantification (**R**, **T**) are shown. Data are presented as mean ± SD; *n* = 3. **U**, **V** Neutral lipid levels detected by BODIPY 493/503 staining in IGF2BP3-knockdown and FASN-overexpressing H1299 cells. Representative fluorescent images (**U**) and quantification (**V**) are shown. Data are presented as mean ± SD; *n* = 3. **W**, **X** Neutral lipid levels measured by Nile Red staining in IGF2BP3-knockdown and FASN-overexpressing H1299 cells. Representative fluorescent images (**W**) and quantification (**X**) are shown. Data are presented as mean ± SD; *n* = 3. *P < 0.05; **P < 0.01; ***P < 0.001; ****P < 0.0001 (two-tailed *t*-test).

To further elucidate the role of the IGF2BP3-FASN axis in NSCLC invasiveness and lipogenesis, we generated IGF2BP3-knockdown and FASN-overexpressing (shIGF2BP3+OE-FASN) H1299 and H157 cell lines. The mRNA level and protein expression were verified by RT-qPCR and immunoblotting (Fig. 6A–D). FASN overexpression significantly rescued the inhibition of cell proliferation (Fig. S8A–D), colony formation (Fig. S8E–G), migration (Fig. 6E–H), and invasion (Fig. 6I–L) induced by IGF2BP3 knockdown, while reducing apoptosis (Fig. S8H–K). Consistently, we found that FASN overexpression effectively rescued IGF2BP3-knockdown mediated decreases in total triglycerides (Fig. 6M, O), total cholesterol (Fig. 6N, P), lipid droplets (Figs. 6Q–T, W, X and S8N, O) and neutral lipid level (Figs. 6U, V and S8L, M) in NSCLC cells. These findings confirm that FASN partly contributes to IGF2BP3-mediated lipid metabolism and invasiveness in NSCLC cells.

**Fig. 7:**
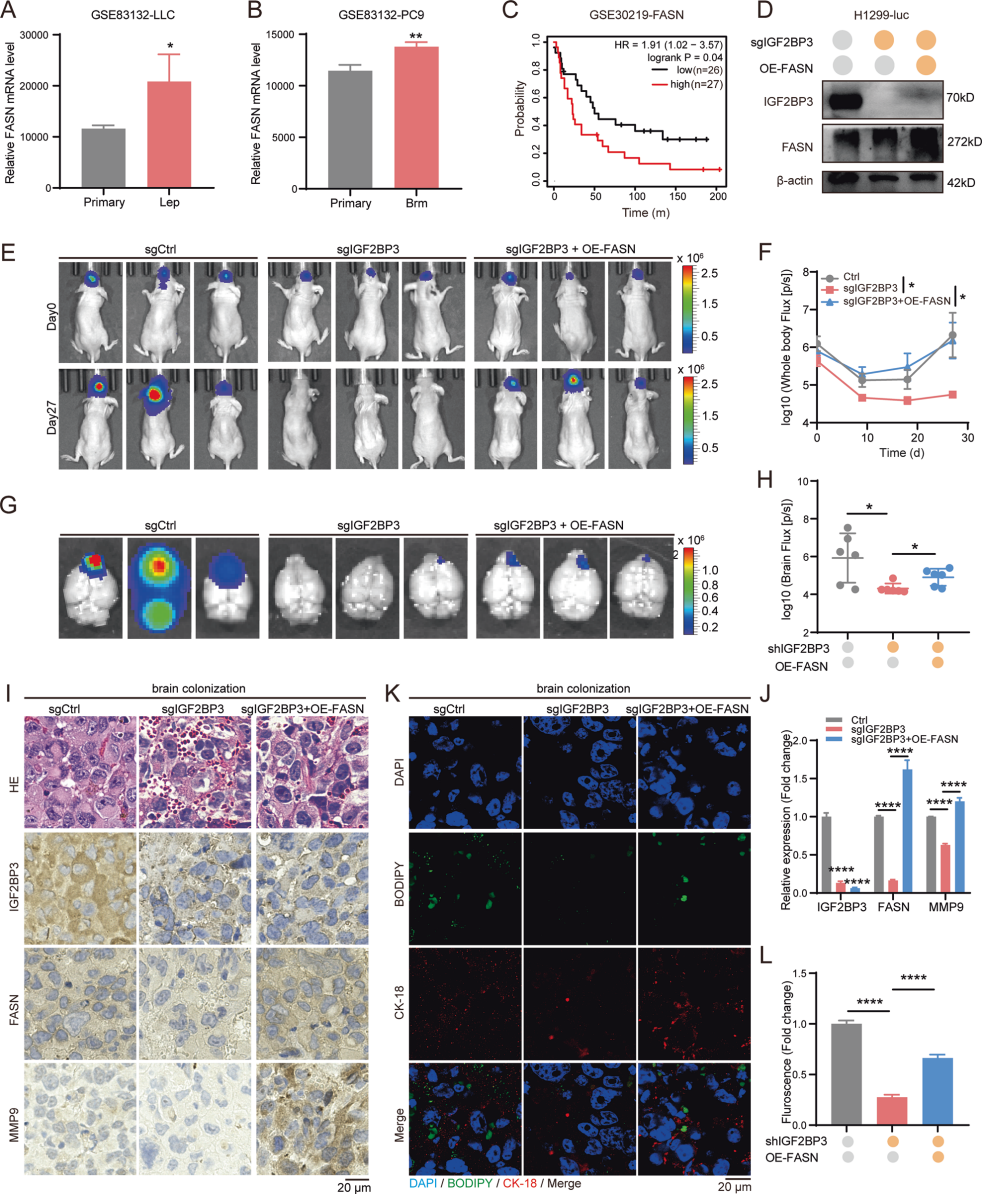
The IGF2BP3-FASN axis mediates lipid metabolism to promote brain colonization in NSCLC. **A** FASN expression was elevated in LLC1-derived leptomeningeal metastatic cells (Lep) compared with primary cells according to GSE83132 dataset. Data are presented as mean ± SD; *n* = 3. **B** FASN expression was elevated in PC9-derived brain metastatic cells (Brm) compared with primary cells according to GSE83132 dataset. Data are presented as mean ± SD; *n* = 3. **C** Overall survival of metastatic lung cancer patients was calculated with the log-rank test and stratified by high or low FASN expression (high: top 50%, *n* = 27; low: bottom 50%, *n* = 26; GSE30219). **D** IGF2BP3 and FASN protein expression detected by Western blotting in IGF2BP3-knockout and FASN-overexpressing H1299-luc cells. **E**, **F** H1299-luc cells (5 × 10^5^) with IGF2BP3 knockout and FASN overexpression were injected into BALB/c-nu/nu mice via stereotactic brain injection (*n* = 6 per group). Representative bioluminescent images of NSCLC xenograft tumor in the brain on day 27 are shown in (**E**). Quantification of bioluminescence on day 27 is shown in (**F**). Data are presented as mean ± SD; *n* = 6. **G**, **H** Representative bioluminescent images of brain tumors on day 27 were captured by the Neurostar IVIS preclinical in vivo imaging system (**G**). Quantification of bioluminescence in brain tumors on day 27 is shown in (**H**). Data are presented as mean ± SD; *n* = 6. **I** H&E staining and IHC analysis of IGF2BP3 and MMP9 in representative brain tumors from IGF2BP3-knockout and FASN-overexpressing mice. Scale bar = 20 μm. **J** Quantification of IGF2BP3 and MMP9 expression levels from IHC analysis in (**I**) using Image Pro Plus. Data are presented as mean ± SD; *n* = 3. **K**, **L** Neutral lipid levels detected by BODIPY 493/503 staining in representative brain tumors from IGF2BP3-knockout and FASN-overexpressing mice. Representative fluorescent images (**K**) and quantification (**L**) are shown. Data are presented as mean ± SD; *n* = 3. *P < 0.05; **P < 0.01; ****P < 0.0001 (two-tailed *t*-test).

Clinically, FASN was upregulated in NSCLC-derived leptomeningeal and brain metastatic tumors compared to primary tumors in the GSE83132 dataset (Fig. 7A, B). Kaplan-Meier survival analysis revealed that high FASN expression correlates with poor prognosis in metastatic lung cancer patients (Fig. 7C). To validate these findings in vivo, we established a mouse stereotactic brain injection model using H1299-luc cells with IGF2BP3 knockout (sgIGF2BP3-luc) or FASN rescue (sgIGF2BP3+OE-FASN-luc), and verified the protein level by immunoblotting (Fig. 7D). H1299-luc cells were injected into the cortex of 4-week-old nude mice and the body weight was measured every three days (Fig. S8P). The tumors were imaged in vivo by IVIS system every nine days and plotted a growth curve. Next, the mice brains were harvested and imaged on day 27. Consistent with the in vitro study, FASN overexpression partly reversed the reduced tumor burden in the brain caused by IGF2BP3 knockout (Fig. 7E–H). IHC staining confirmed reduced IGF2BP3 and increased FASN expression in brain colonization tumor, with FASN rescue restoring MMP9 expression (Fig. 7I–J). Immunofluorescence staining further demonstrated that FASN overexpression rescued neutral lipid production in brain colonization tumor (Fig. 7K, L). Collectively, these results highlight that the IGF2BP3-FASN axis is a potential therapeutic target for regulating the growth of NSCLC cells in the brain through lipid metabolism.

## Discussion

In this study, we elucidated the critical role of lipid metabolism in driving the growth of NSCLC cells in the brain through the IGF2BP3-FASN axis. IGF2BP3, initially identified as an oncofetal protein [39–41], is overexpressed in various cancers and has been implicated in tumor proliferation, drug resistance, and metastasis [29, 42–44]. NSCLC patients frequently develop brain metastases during disease progression, yet current therapeutic options remain limited, underscoring the need for novel therapeutic targets. Here, we demonstrated that IGF2BP3 is a potential therapeutic target for NSCLC brain colonization, acting through FASN-mediated lipid metabolism. IGF2BP3 is upregulated in NSCLC brain metastases and positively correlates with key epithelial-mesenchymal transition (EMT) genes, as supported by bioassays and bioinformatics analyses. Elevated IGF2BP3 expression is associated with poor clinical prognosis and advanced metastatic stages, highlighting its important role in tumor metastasis and its potential as a therapeutic target. Consistent with our findings, recent studies have linked IGF2BP3 to brain metastasis in breast cancer [34], further supporting its role in tumor dissemination to the brain. However, the mechanisms that IGF2BP3 mediates brain colonization of NSCLC cells remain incompletely understood.

Our functional studies revealed that targeting IGF2BP3 significantly inhibits invasiveness in vitro and reduces NSCLC tumor burden in the brain in vivo. IGF2BP3 knockdown suppressed NSCLC cell proliferation, migration, and invasion while promoting apoptosis. Using a mouse stereotactic brain injection model, we found that IGF2BP3 knockout significantly reduced NSCLC tumor burden in the brain, whereas IGF2BP3 overexpression exacerbated brain colonization. These findings suggest that IGF2BP3 is a promising therapeutic target for NSCLC brain metastasis.

To uncover the mechanisms by which IGF2BP3 promotes the growth of NSCLC cells in the brain, we employed RNA sequencing and lipidomics analyses. Gene enrichment analysis highlighted metabolic and lipogenesis pathways as key downstream targets of IGF2BP3. The brain microenvironment imposes unique metabolic constraints on cancer cells, and recent studies have demonstrated that metabolic adaptations, such as de novo serine synthesis and acetate utilization, are critical for tumor survival in the brain. Lipid metabolism, in particular, plays a pivotal role in brain metastasis. For example, Ngo and colleagues found that tumor cells require de novo serine synthesis to proliferate in the brain microenvironment in breast cancer brain metastases [9], which has reduced amino acid levels relative to plasma [45, 46]. Mashimo and colleagues demonstrated that brain metastasis showed special acetate adaptation for meeting the high biosynthetic and bioenergetic demands in glioblastoma [47]. It was reported that fatty acid synthesis is required for breast cancer brain metastasis [17]. Consistently, lipid synthesis was more active in brain metastasis in breast cancer [48]. These studies suggested that the brain microenvironment can impose metabolic constraints on cancer cells and that identifying metabolic limitations could inform strategies to overcome brain metastasis, especially lipid metabolism. Our lipidomics analysis revealed that IGF2BP3 knockdown downregulates various lipid metabolites, including fatty acids and triglycerides. Recent study highlighted that IGF2BP3 regulate Stearoyl-CoA Desaturase (SCD), a key enzyme in lipogenesis, to affect the metabolic pathway in cervical cancer brain metastasis [32]. Consistent with these findings, IGF2BP3 depletion reduced neutral lipid accumulation, total triglycerides, cholesterol levels, and lipid droplet formation in NSCLC cells. These results underscore the role of IGF2BP3 in regulating lipogenesis and fatty acid metabolism in NSCLC.

We further investigated the mechanism by which IGF2BP3 modulates fatty acid metabolism. Overlapping RNA-seq and RIP-seq data identified FASN, a key enzyme in lipogenesis, as a downstream target of IGF2BP3. FASN is central to fatty acid metabolism [49] and mediates lipogenesis to facilitated the growth of a variety of human cancers [13, 50]. Recent study also showed that FASN activating fatty acid metabolism to promote cancer metastasis in male breast cancer [16]. In our study, FASN was essential for IGF2BP3-mediated lipid metabolism and brain colonization. IGF2BP3 knockdown significantly reduced FASN expression at both mRNA and protein levels and decreased FASN mRNA stability. Mechanistically, IGF2BP3 directly binds to FASN mRNA, stabilizing it in an RNA-binding-dependent manner. Clinically, FASN is upregulated in leptomeningeal and brain metastatic tumors and correlates with poor prognosis in NSCLC patients. These findings align with previous studies linking FASN to metastasis in osteosarcoma and colorectal cancer [51, 52].

To determine whether FASN is the crucial downstream effector of IGF2BP3-mediated brain metastasis, we overexpressed FASN in IGF2BP3-knockdown NSCLC cells. FASN overexpression partly rescued the phenotypes of reduced growth, invasiveness, and neutral lipid accumulation caused by IGF2BP3 depletion. Furthermore, we generated a mouse stereotactic brain injection model based on NSCLC cells. FASN overexpression effectively alleviated IGF2BP3-dependent brain colonization and restored lipid droplet formation in a mouse stereotactic brain injection model. These results suggest that the IGF2BP3-FASN axis plays a critical role in the growth of NSCLC cells in the brain microenvironment. While FASN overexpression partially rescued IGF2BP3-knockdown effects on lipid metabolism and tumor burden in brain, the incomplete restoration implies additional downstream mediators or mechanisms may also contribute. Furthermore, it is undeniable that our model focuses specifically on the later stages of metastasis (colonization of NSCLC cells in the brain microenvironment), rather than the entire metastatic process. In the future, further investigation and validation of these findings using spontaneous metastasis models (intracardiac or orthotopic injection) will enrich and deepen the understanding of the role of the IGF2BP3-FASN axis in brain metastasis of NSCLC. Notwithstanding these limitations, this study does suggest the potential of IGF2BP3 as a drive gene for NSCLC cells to grow in the brain.

In summary, our study identifies IGF2BP3 as a key regulator of the growth of NSCLC cells in the brain microenvironment through lipid metabolism. IGF2BP3 is upregulated in NSCLC brain metastases and promotes FASN-mediated neutral lipid accumulation. Mechanistically, IGF2BP3 binds to FASN mRNA, enhancing its stability in an RNA-binding-dependent manner. The IGF2BP3-FASN axis emerges as a critical regulator of brain colonization, linking lipid metabolism to tumor dissemination. These findings highlight IGF2BP3 as a potential therapeutic target for inhibiting the growth of NSCLC cell in the brain.

## Materials and methods

### Cell culture

The H1299, H157 and HEK-293T cell lines were obtained from the American Type Culture Collection (ATCC, USA). The H1299 and H157 cell lines were cultured in RPMI-1640 (Gibco, USA) supplemented with 10% FBS (Gibco, USA) and 1% penicillin-streptomycin (PS, Gibco, USA). HEK-293T cells were cultured in DMEM (Gibco, USA) with 10% FBS and 1% PS. All cell lines were cultivated at 37 °C under 5% CO₂. To minimize phenotypic changes, cells were aliquoted into multiple vials upon receipt and routinely tested for mycoplasma contamination using the Mycoplasma Stain Assay Kit (C0296, Beyotime, China). Cell line authentication was performed using STR analysis with the GenePrint 10 System (B9510, Promega, USA) following the manufacturer’s instructions.

### Plasmid construction, cell transfection, and virus infection

Stable knockout of target genes was achieved using the lentiCRISPRv2 system, while stable knockdown was accomplished via lentiviral delivery of specific short-hairpin RNAs (shRNAs). Primer sequences for vector construction are listed in Table S1.

For overexpression studies, human IGF2BP3 cDNA (NM_006547) and FASN cDNA (NM_004104.5) were cloned into the pCDNA3.1 lentiviral vector (CD510B-1, System Biosciences).

### Antibodies

The following antibodies were used: anti-IGF2BP3 (WB 1:1000; ab179807, Abcam, USA), anti-IGF2BP3 (IHC 1:100; EM1701-15, HuaBio, China), anti-FASN (WB 1:500; IHC 1:100; 66591-1-Ig, Proteintech, China), anti-β-actin (WB 1:1000, #4967, Cell Signaling Technology, USA), anti-MMP9 (IHC 1:500; GB11132, Servicebio, China), and anti-Cytokeratin 18 (IF 1:200; GB11232, Servicebio, China).

### Western blotting

Cells were lysed in 100 μL modified RIPA buffer (P0013B, Beyotime, China) containing protease inhibitors (#11836153001, Roche, Switzerland) and phosphatase inhibitors (Bimake, USA). Protein concentrations were determined using the BCA protein assay kit (23252, ThermoFisher, USA). Proteins were separated by 8–10% SDS-PAGE and transferred to polyvinylidene fluoride (PVDF) membranes (Millipore, USA). Membranes were blocked with 5% BSA and probed with primary and secondary antibodies. Target proteins were visualized using a chemiluminescence imaging system (1708280, Bio-Rad, USA).

### Immunohistochemistry

Tissue slides were deparaffinized, rehydrated, and subjected to antigen retrieval in sodium citrate buffer. Tumor sections were blocked with 5% goat serum containing 0.1% Triton X-100 and 3% H₂O₂ in PBS for 60 min at room temperature, followed by incubation with primary antibodies at 4 °C overnight. IHC staining was performed using horseradish peroxidase (HRP)-conjugated secondary antibodies and DAB detection. Nuclei were counterstained with DAPI, and images were acquired using an imaging system (AMAFD1000, ThermoFisher, USA).

### Cell proliferation assays

For the CCK-8 assay, cells were seeded at 1000 cells/well in 96-well plates. Cell viability was assessed at 0, 24, 48, 72, 96, and 120 h using the CCK-8 reagent. Absorbance was measured at 450 nm using a microplate reader (FLUOstar, Omega, USA) after a 2-h incubation at 37 °C. For cell counting assays, cells were seeded at 5 × 10^4^ cells/well in 6-well plates, and cell numbers were counted at the indicated time points.

### Colony formation assay

Cells were seeded in 6-well plates at densities of 500 cells/well (H1299) or 1 × 10^3^ cells/well (H157) and cultured for 10 days. Colonies were fixed with 4% paraformaldehyde, stained with crystal violet (Beyotime, China), and imaged.

### Cell apoptosis assay

Apoptosis was assessed using the Annexin V-PI Apoptosis Detection Kit I (BB-4101, BestBio, China). Briefly, cells were collected, centrifuged at 1000 × *g* for 3 min, and resuspended in 400 μL annexin V-FITC binding buffer containing 5 μL annexin V-FITC and 10 μL propidium iodide. Samples were analyzed by flow cytometry (CytoFLEX, Beckman, USA) and quantified using FlowJo V10 software.

### Wound healing assay

Cells were seeded in 6-well plates and grown to 90% confluence. A sterile pipette tip was used to create a scratch, and detached cells were removed by washing with PBS. Cells were incubated in 2% FBS medium for 24 h, and images were captured at 0 and 24 h. The rate of wound healing = [(the wound width of 0 h–48 h)/0 h wound width] × 100%.

### Transwell invasion

Transwell chambers (Thermo Fisher Scientific) were used for invasion assays. Cells were seeded in the upper chamber in 100 μL of medium containing 10% FBS, while the lower chamber contained medium with 20% FBS as a chemoattractant. After 24 h, non-invading cells were removed, and invading cells were fixed with 4% paraformaldehyde, stained with 1% crystal violet, and imaged using an EVOS M5000 Fluorescence Microscope.

### Measurement of TG and TC

Total triglycerides (TG) and total cholesterol (TC) levels were measured using the Total Triglycerides Assay Kit (BB-47436, Bestbio) and Total Cholesterol Assay Kit (BB-47435, Bestbio), respectively. Cells were washed with isotonic buffer, lysed in PBS containing 3% Triton X-100, and centrifuged. TG or TC assay reagents were added to the lysates, and absorbance was measured at 520 nm using a microplate reader. Concentrations were calculated according to the manufacturer’s protocols.

### Bodipy and Nile Red assays

Cells were fixed with 4% formaldehyde. For BODIPY staining, cells were incubated in PBS for 30 min. For Nile Red staining, cells were incubated in PBS for 1 h. After washing with PBS, nuclei were counterstained with DAPI. Fluorescence was visualized using an FV-1000/ES confocal microscope (Olympus, Japan) or quantified by flow cytometry.

### Quantitative RT-qPCR assay

Total RNA was extracted using Trizol reagent (15596026, Invitrogen, USA) and treated with DNase I to ensure RNA quality. cDNA synthesis and quantitative PCR were performed using SYBR PCR Master Mix (Yeasen, China). Primer sequences are listed in Table S2.

### Metabolomics analysis

H1299 and H1299 shIGF2BP3 cells (1 × 10^7^ cells) were homogenized in 1 mL of a methanol:ddH_2_O solution (4:1, v/v) and centrifuged at 12,000×*g* at 4 °C for 10 min. The supernatant was collected, dried using a vacuum concentrator, and reconstituted in 300 μL of acetonitrile:0.1% formic acid (FA) solution (1:9, v/v, pre-chilled at −20 °C). After filtration through a 0.22 μm membrane, the filtrate was transferred to a detection vial. Chromatographic separation was performed using a Thermo Ultimate 3000 system equipped with an ACQUITY UPLC® HSS T3 column (150 × 2.1 mm, 1.8 μm, Waters) maintained at 40 °C. The autosampler temperature was set to 8 °C. Gradient elution was achieved using 0.1% FA in water (solvent C) and 0.1% FA in acetonitrile (solvent D) or 5 mM ammonium formate in water (solvent A) and acetonitrile (solvent B) at a flow rate of 0.25 mL/min. Each sample (2 μL) was injected after equilibration. The gradient program was as follows: 0–1 min, 2% B/D; 1–9 min, 2–50% B/D; 9–12 min, 50–98% B/D; 12–13.5 min, 98% B/D; 13.5–14 min, 98–2% B/D; 14–20 min, 2% D (positive mode) or 14–17 min, 2% B (negative mode). ESI-MSn experiments were conducted on a Thermo Q Exactive mass spectrometer with spray voltages of 3.8 kV (positive mode) and −2.5 kV (negative mode). Sheath and auxiliary gases were set to 45 and 15 arbitrary units, respectively, and the capillary temperature was maintained at 325 °C. The Orbitrap analyzer scanned a mass range of m/z 81–1000 at a resolution of 70,000. Data-dependent acquisition (DDA) MS/MS experiments were performed using higher-energy collisional dissociation (HCD) with a normalized collision energy of 30 eV. Dynamic exclusion was applied to remove redundant data. LC-MS detection and analysis were performed by Wininnovate Bio, LTD (Shenzhen, China).

### RNA immunoprecipitation sequencing

RNA immunoprecipitation (RIP) assays were performed using the Magna RIP™ RNA-Binding Protein Immunoprecipitation Kit (17–700, Merck Millipore, USA) according to the manufacturer’s protocol. Briefly, stable IGF2BP3-overexpressing H1299 cells were lysed in complete RIP lysis buffer. Anti-IGF2BP3 mouse monoclonal antibody (EM1701-15, HuaBio, China) and anti-IgG mouse monoclonal antibody were conjugated to magnetic beads to immunoprecipitate RNA-protein complexes. Enriched mRNAs were validated by RT-qPCR using RIP-specific primers.

### RNA stability assay (half-life detection)

RNA stability was assessed by treating cells with 20 μg/mL actinomycin D (Act-D, HY-17559, MCE, USA). Cells were collected at 0, 1, 3, 6, 9, 16, and 24-h post-treatment, and RNA was isolated for RT-qPCR analysis.

### Animal experiments

All animal experiments were approved by the Animal Ethics Committee of Sun Yat-Sen University and conducted in accordance with the Guide for the Care and Use of Laboratory Animals. Four-week-old male BALB/c nude mice (18 g, SPF grade) were obtained from the Experimental Animal Center of Sun Yat-sen University (Guangdong, China).

For the subcutaneous tumor model, H1299 cells (5 × 10^6^) were injected into the nude mice. Tumor growth was monitored for 27 days until harvested. At the endpoint, mice were euthanized, and subcutaneous tumor were harvested for histological analysis.

For the stable sgIGF2BP3 stereotactic brain tumor model, sgCtrl or sgIGF2BP3 H1299 cells (5×10⁵) were injected into the cortex of nude mice. For the IGF2BP3 overexpression model, OE-Ctrl or OE-IGF2BP3 H1299 cells (5 × 10^5^) were injected. For rescue experiments, sgCtrl, sgIGF2BP3, or sgIGF2BP3&OE-FASN H1299 cells (5 × 10^5^) were injected. Tumor growth was monitored using the Neurostar IVIS preclinical in vivo imaging system every 9 days lasted for a total of 27 days. At the endpoint, mice were euthanized, and brains were harvested for histological analysis.

### Bioinformatics analysis

Gene expression profiles (TCGA-LUAD, GSE11049, GSE83132, GSE33532, GSE198291, GSE90639, GSE79147, GSE230646, GSE231938, and GSE30219) were downloaded from the Gene Expression Omnibus (GEO) database and The Cancer Genome Atlas (TCGA, http://cancergenome.nih.gov). The online database R2 Genomics Analysis and Visualization Platform (https://hgserver1.amc.nl/cgi-bin/r2/main.cgi) was applied to determine the correlation of the related genes in GSE33532. Pearson analysis was applied to analyze the correlation of the related gene in GSE198291. Differentially expressed genes (fold change > 1.5, *p* < 0.05) and RIP data (fold change > 2, *p* < 0.05) were analyzed. Kaplan-Meier Plotter (https://kmplot.com/analysis/) was used to determine the clinical survival of the related genes (cutoff: 50%; *p* < 0.05). KEGG pathway enrichment analysis was performed using DAVID Bioinformatics Resources 6.8 (http://david.ncifcrf.gov). Metabolomics data were enriched and differential metabolites (Fold change > 1.5; *p* < 0.05) were analyzed using the MetaboAnalyst database (https://www.metaboanalyst.ca/home.xhtml).

### Statistical analysis

Statistical analysis was performed by GraphPad Prism 8.0 software (GraphPad, Inc., La Jolla, CA, USA) and quantitative data were presented as the mean ± SD unless stated otherwise. Statistical differences were assessed using a two-tailed Student’s *t*-test. The sample sizes (*n*) and probability (*p*) values for each experiment were indicated in detail in figure legends. Survival curves were constructed using the Kaplan–Meier method and analyzed by the log-rank test. The correlation analysis was assessed by Pearson’s correlation analysis. The detail of colors and the node size for indicated graph were indicated in figure legends. To represent the results as a heat map, the *z*-score values were shown with the color intensity, and the remaining values were ranked according to their relative values. The graphic abstract was drawn by Figdraw (https://www.figdraw.com). The schematics of mice cortex stereotactic injection and in vivo imaging system was drawn by Biorender (https://biorender.com). Differences between values were considered statistically significant when *p < 0.05, **p < 0.01, ***p < 0.001, and ****p < 0.0001.

## Supplementary information

original western blot

Supplementary materials

## Data Availability

All data in the study were included in the article or uploaded as supplemental online information. All unique reagents generated relevant to this study and the raw data associated with this paper are available from the corresponding authors with a complete materials transfer agreement. RNA-Seq data has been deposited to GEO under the following accession number: GSE298476.
