# Supplementary figures and images for: The IGF2BP3–FASN axis drives lipid metabolic reprogramming to promote brain colonization in non-small cell lung cancer
Source: Cell Death Dis. 2025 Oct 6;16(1):684. doi: 10.1038/s41419-025-08006-z (PMC12501377; doi:10.1038/s41419-025-08006-z)

Fig 5.E

H1299

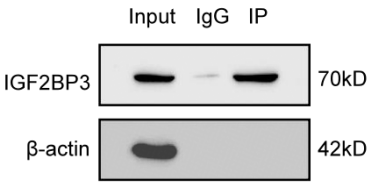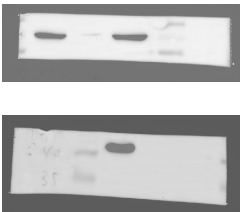

Fig 5.J

H1299

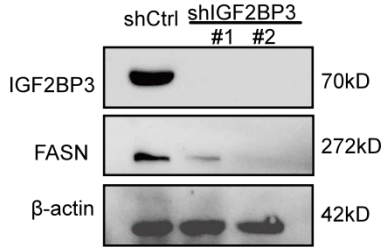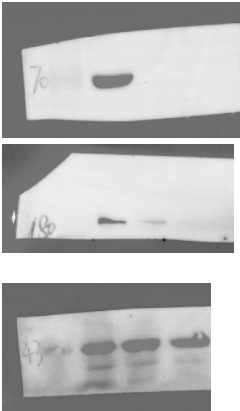

Fig 5.K

H157

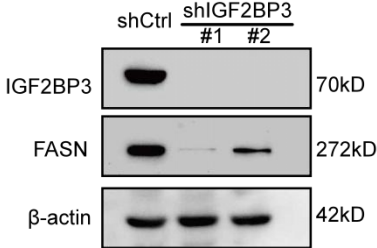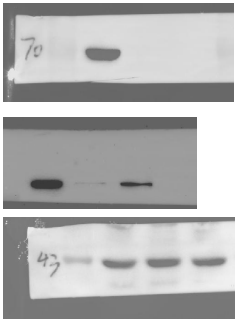

Fig 5.N

H1299

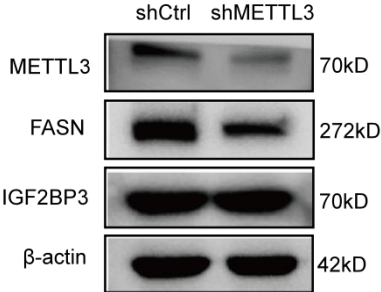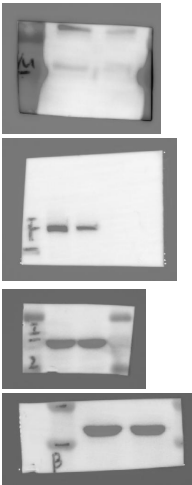

Fig 6.C

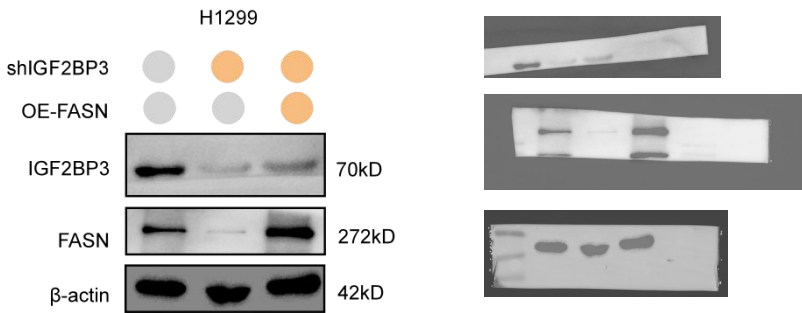

Fig 6.D

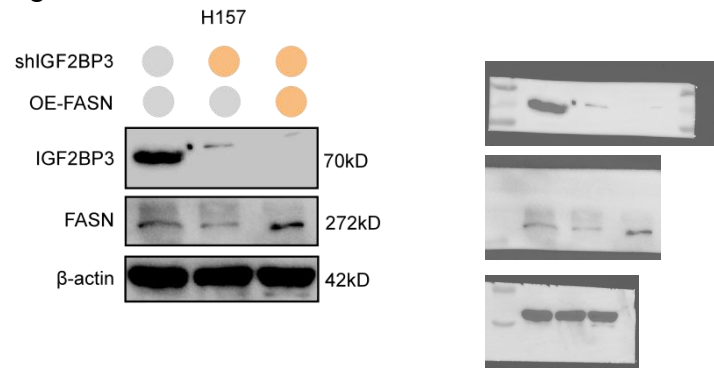

Fig 7.D

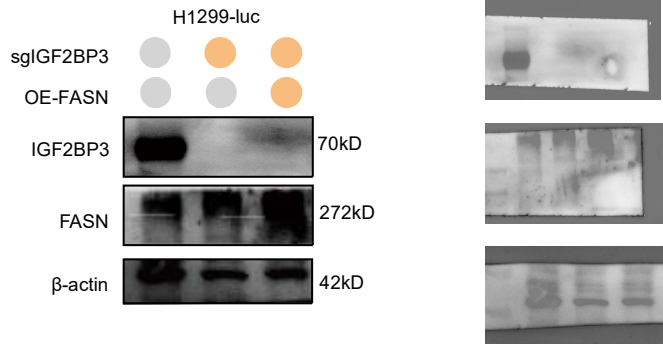

Fig S1.C

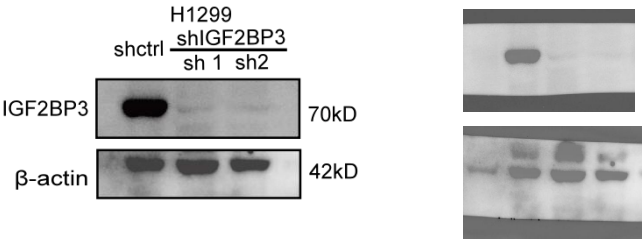

Fig S1.D

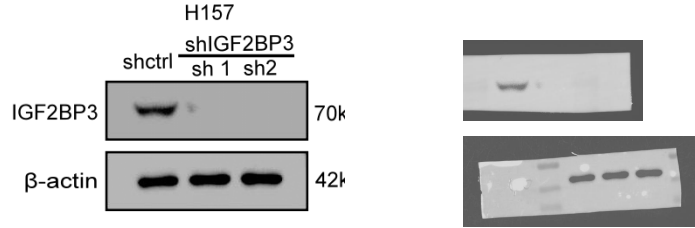

Fig S1.L

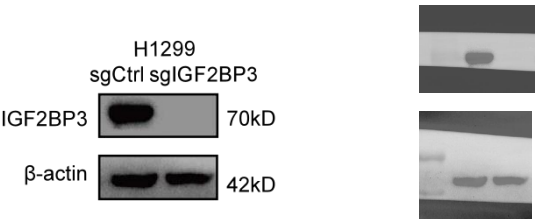

Fig S3.C

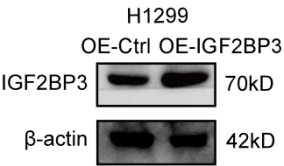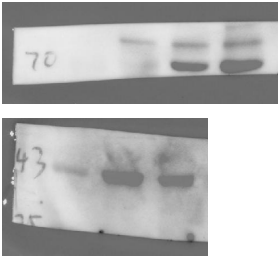

Fig S3.D

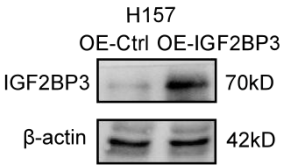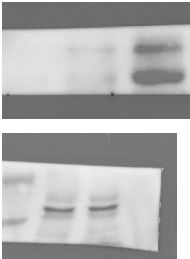

Fig S3.P

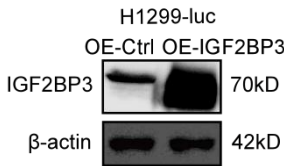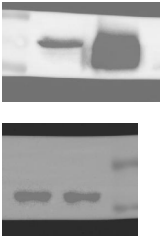

Fig S5.D

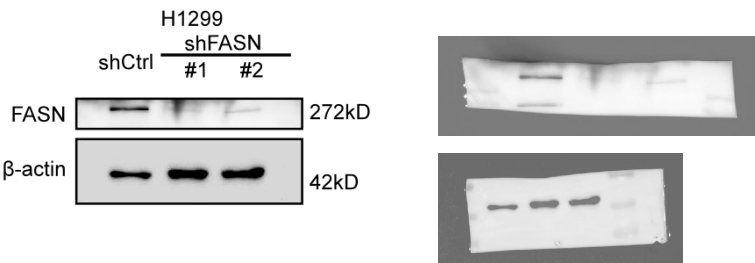

Fig S5.E

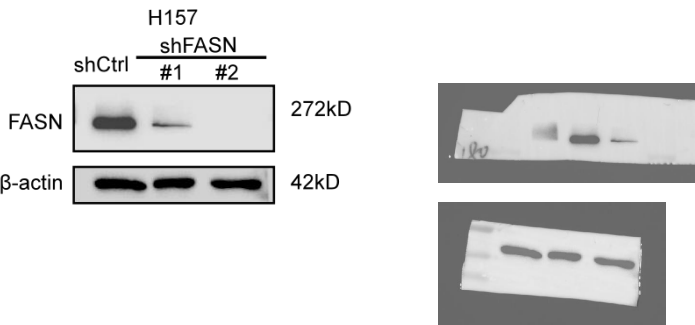

Supplement: Supplementary file 1 — original western blot [file 41419_2025_8006_MOESM1_ESM.pdf]
