# Supplementary material for: The IGF2BP3–FASN axis drives lipid metabolic reprogramming to promote brain colonization in non-small cell lung cancer
Source: Cell Death Dis. 2025 Oct 6;16(1):684. doi: 10.1038/s41419-025-08006-z (PMC12501377; doi:10.1038/s41419-025-08006-z)
Supplement: Supplementary file 2 — Supplementary materials [file 41419_2025_8006_MOESM2_ESM.docx]

**Supplementary materials for**

**The IGF2BP3-FASN Axis Drives Lipid Metabolic Reprogramming to Promote Brain Colonization in Non-Small Cell Lung Cancer**

Jingwei Li^#1^, Shumin Ouyang^#1^, Ziyou Lin^#1,2^, Keren Peng^1^, Jiayu Yan^1^, Minyuan Lu^1^, Wen Ding^1,3^, Jianshan Mo^1^, Yingxue Su^3^, Libin Wang^4^, Peibin Yue^5^, JianJin Lu^6^, Xiangchao, Yao^3*^, Yandong Wang^3*^, Xiaolei Zhang^1*^

1. National-Local Joint Engineering Laboratory of Druggability and New Drug Evaluation, Guangdong Key Laboratory of Chiral Molecule and Drug Discovery, School of Pharmaceutical Sciences, Sun Yat-sen University, Guangzhou, 510006, China
2. State Key Laboratory of Oncology in South China, Guangdong Provincial Clinical Research for Cancer, Sun Yat-sen University Cancer Center, Guangzhou, 510060, China
3. State Key Laboratory of Ophthalmology, Zhongshan Ophthalmic Center, Sun Yat-sen University, Guangzhou, 510060, China
4. Huazhong University of Science and Technology Union Shenzhen Hospital, Shenzhen, 518052, China
5. Department of Medicine, Division of Hematology-Oncology, and Samuel Oschin Comprehensive Cancer Institute, Cedars-Sinai Medical Center, Los Angeles, CA, 90048, USA
6. State Key Laboratory of Quality Research in Chinese Medicine, Institute of Chinese Medical Sciences, University of Macau, Macao, China

^#^These authors contributed equally to this work.

*To whom correspondence should be addressed:

E-mail:

zhangxlei5@mail.sysu.edu.cn (Xiaolei Zhang), wangydsyj@163.com (Yandong Wang)

yxc129@163.com (Xiangchao Yao)

**Supplementary files:**

**Supplementary figures S1-S8**

**Supplementary tables S1-S3**

**Original western blots**

**Figure S1. IGF2BP3 promotes proliferation and colonization in NSCLC**

**A-B**. The mRNA expression of IGF2BP3 was evaluated by RT-qPCR in IGF2BP3-knockdown H1299 and H157 cells. Data are presented as mean ± SD; n=3.

**C-D**. The protein expression of IGF2BP3 was evaluated by Western Blotting in IGF2BP3-knockdown H1299 and H157 cells.

**E-F**. Cell proliferation was evaluated by CCK8 assay in IGF2BP3-knockdown H1299 and H157 cells for 0, 24, 48, 72 and 96 h. Data are presented as mean ± SD; n=3.

**G-H**. Cell proliferation was evaluated by cell counting assay in IGF2BP3-knockdown H1299 and H157 cells for 0, 24, 48, 72 and 96 h. Data are presented as mean ± SD; n=3.

**I-K**. Colony survival was detected by colony formation assay in IGF2BP3-knockdown H1299 and H157 cells for 10 days. The representative images are shown in (**I**) and quantification are shown in (**J** and **K**). Data are presented as mean ± SD; n=3.

**L**. The protein expression level of IGF2BP3 was detected by Western Blotting in IGF2BP3-knockout H1299-luc cells (luc: luciferase).

**M-P**. Cell apoptosis was analyzed by flow cytometry in IGF2BP3-knockdown H1299 and H157 cells. The representative images are shown in (**M** and **O**) and quantification are shown in (**N** and **P**). Data are presented as mean ± SD; n=3.

*P < 0.05; **P < 0.01; ***P < 0.001; ****P < 0.0001; two-tailed t-test.

**Figure S2. IGF2BP3 promotes migration and invasion in NSCLC**

**A**. Cell migration determined by wound healing assay in H157 cells treated with shRNAs against IGF2BP3 or control for 24 h. Scale bars = 50 μm.

**B**. Quantification of migration ability of IGF2BP3-knockdown H157 cells in (**A**) was analyzed by Image J. Data are presented as mean ± SD; n=3.

**C**. Cell invasion determined by transwell assay in H157 cells treated with shRNAs against IGF2BP3 or control for 24 h. Scale bars = 50 μm.

**D**. Quantification of invasion ability of IGF2BP3-knockdown H157 cells in (**C**) was analyzed by Image J. Data are presented as mean ± SD; n=3.

**E**. Cell migration determined by wound healing assay in H157 cells treated with IGF2BP3 overexpression or control for 24 h. Scale bars = 50 μm.

**F**. Quantification of migration ability of IGF2BP3-overexpression H157 cells in (E) was analyzed by Image J. Data are presented as mean ± SD; n=3.

**G**. Cell invasion determined by transwell assay in H157 cells treated with IGF2BP3 overexpression or control for 24 h. Scale bars = 50 μm.

**H**. Quantification of invasion ability of IGF2BP3-overexpression H157 cells in (G) was analyzed by Image J. Data are presented as mean ± SD; n=3.

**I**. Schematics of mice cortex stereotactic injection and *in vivo* imaging system by Biorender (<https://biorender.com>).

**J**. The mice body weight in IGF2BP3-knockout H1299 cells orthotopic implantation model was measured every three days. Data are presented as mean ± SD; n=6.

**K**. The mice body weight in IGF2BP3-overexpression H1299 cells orthotopic implantation model was measured every three days. Data are presented as mean ± SD; n=6.

**L**. Quantification for bioluminescent images of brain metastasis mouse model in IGF2BP3-knockout group on 0, 9, 18 and 27 days after modeling. Data are presented as mean ± SD; n=6.

**M**. Quantification for bioluminescent images of brain metastasis mouse model in IGF2BP3-overexpression group on 0, 9, 18 and 27 days after modeling. Data are presented as mean ± SD; n=6.

ns, no significant; *P < 0.05; **P < 0.01; ***P < 0.001; ****P < 0.0001; two-tailed t-test.

**Figure S3. IGF2BP3 promotes proliferation and colonization in NSCLC**

**A-B**. The mRNA expression level of IGF2BP3 was evaluated by RT-qPCR in IGF2BP3-overexpression H1299 and H157 cells. Data are presented as mean ± SD; n=3.

**C-D**. The protein expression level of IGF2BP3 was evaluated by Western Blotting in IGF2BP3-overexpression H1299 and H157 cells.

**E-F**. Cell proliferation was evaluated by CCK8 assay in IGF2BP3-overexpression H1299 and H157 cells for 0, 24, 48, 72, 96 and 120 h. Data are presented as mean ± SD; n=3.

**G-H**. Cell proliferation was evaluated by cell counting assay in IGF2BP3-overexpression H1299 and H157 cells for 0, 24, 48, 72, 96 and 120 h. Data are presented as mean ± SD; n=3.

**I-K**. Colony survival was detected by colony formation assay in IGF2BP3-overexpression H1299 and H157 cells for 10 days. The representative images are shown in (**I**) and quantification are shown in (**J** and **K**). Data are presented as mean ± SD; n=3.

**L-O**. Cell apoptosis was analyzed by flow cytometry in IGF2BP3-overexpression H1299 and H157 cells. The representative images are shown in (**L** and **N**) and quantification are shown in (**M** and **O**). Data are presented as mean ± SD; n=3.

**P**. The protein expression level of IGF2BP3 was detected by Western Blotting in IGF2BP3-overexpression H1299-luc cells.

*P < 0.05; **P < 0.01; ***P < 0.001; ****P < 0.0001; two-tailed t-test.

**Figure S4. IGF2BP3 regulates lipid metabolism in NSCLC *in vitro***

**A**. Total triglycerides assay kit showing total triglycerides level was decreased in IGF2BP3-knockdown H157 cells compared with control cells. Data are presented as mean ± SD; n=3.

**B**. Total cholesterol assay kit showing total cholesterol level was decreased in IGF2BP3-knockdown H157 cells compared with control cells. Data are presented as mean ± SD; n=3.

**C**. Total Triglycerides Assay Kit showing total triglycerides level was evaluated in IGF2BP3-overexpression H157 cells compared with control cells. Data are presented as mean ± SD; n=3.

**D**. Total cholesterol assay kit showing total cholesterol level was evaluated in IGF2BP3-overexpression H157 cells compared with control cells. Data are presented as mean ± SD; n=3.

**E-F**. Neutral lipid levels were detected by BODIPY 493/503 probe in IGF2BP3-knockdown H157 cells examined by microscope. The representative fluorescent images (**E**) and quantification (**F**) were shown. Data are presented as mean ± SD; n=3.

**G-H**. Neutral lipid levels were measured by Nile Red in IGF2BP3-knockdown H157 cells examined by microscope. The representative fluorescent images (**G**) and quantification (**H**) were shown. Data are presented as mean ± SD; n=3.

**I-J**. Neutral lipid levels were detected by BODIPY 493/503 probe in IGF2BP3-overexpression H157 cells examined by microscope. The representative fluorescent images (**I**) and quantification (**J**) were shown. Data are presented as mean ± SD; n=3.

**K-L**. Neutral lipid levels were measured by Nile Red in IGF2BP3-overexpression H157 cells examined by microscope. The representative fluorescent images (**K**) and quantification (**L**) were shown. Data are presented as mean ± SD; n=3.

**M-N**. Neutral lipid levels were analyzed by flow cytometry in IGF2BP3-knockdown H157 cells treated with Nile Red. The representative images are shown in (**M**) and quantification are shown in (**N**). Data are presented as mean ± SD; n=3.

**O-P**. Neutral lipid levels were analyzed by flow cytometry in IGF2BP3-overexpression H157 cells treated with Nile Red. The representative images are shown in (**O**) and quantification are shown in (**P)**. Data are presented as mean ± SD; n=3.

*P < 0.05; **P < 0.01; ***P < 0.001; ****P < 0.0001; two-tailed t-test.

**Figure S5. FASN promotes proliferation and brain colonization in NSCLC**

**A**. FASN mRNA level evaluated by RT-qPCR in stable IGF2BP3-konckdown and METTL3-overexpression NSCLC cells. Data are presented as mean ± SD; n=3.

**B-C**. The mRNA expression level of FASN was evaluated by RT-qPCR in FASN-knockdown H1299 and H157 cells. Data are presented as mean ± SD; n=3.

**D-E**. The protein expression level of FASN was evaluated by Western Blotting in FASN-knockdown H1299 and H157 cells.

**F-G**. Cell proliferation was evaluated by CCK8 assay in FASN-knockdown H1299 and H157 cells for 0, 24, 48, 72, 96 and 120 h. Data are presented as mean ± SD; n=3.

**H-I**. Cell proliferation was evaluated by cell counting assay in FASN-knockdown H1299 and H157 cells for 0, 24, 48, 72, 96 and 120 h. Data are presented as mean ± SD; n=3.

**J-L**. Colony survival was detected by colony formation assay in FASN-knockdown H1299 and H157 cells for 10 days. The representative images are shown in (**J**) and quantification are shown in (**K** and **L**). Data are presented as mean ± SD; n=3.

**M-P**. Cell apoptosis was analyzed by flow cytometry in FASN-knockdown H1299 and H157 cells. The representative images are shown in (**M** and **O**) and quantification are shown in (**N** and **P**). Data are presented as mean ± SD; n=3.

*P < 0.05; **P < 0.01; ***P < 0.001; ****P < 0.0001; two-tailed t-test.

**Figure S6. FASN promotes NSCLC migration, invasion and lipogenesis in NSCLC**

**A**. Cell invasion determined by transwell assay in H1299 cells treated with shRNAs against FASN or control for 24 h. Scale bars = 50 μm.

**B**. Quantification of cell invasion of FASN-knockdown H1299 cells in (**A**) was analyzed by Image J. Data are presented as mean ± SD; n=3.

**C**. Cell migration determined by wound healing assay in H1299 cells treated with shRNAs against FASN or control for 24 h. Scale bars = 50 μm.

**D**. Quantification of migration ability of FASN-knockdown H1299 cells in (**C**) was analyzed by Image J. Data are presented as mean ± SD; n=3.

**E**. Total triglycerides assay kit showing total triglycerides level was evaluated in FASN-knockdown H1299 cells compared with control cells. Data are presented as mean ± SD; n=3.

**F**. Total cholesterol assay kit showing total cholesterol level was evaluated in FASN-knockdown H1299 cells compared with control cells. Data are presented as mean ± SD; n=3.

**G-H**. Neutral lipid levels were analyzed by flow cytometry in FASN-knockdown H1299 cells treated with Nile Red. The representative images are shown in (**G**) and quantification are shown in (**H**). Data are presented as mean ± SD; n=3.

**I-J**. Neutral lipid levels were detected by BODIPY 493/503 probe in FASN-knockdown H1299 cells examined by microscope. The representative fluorescent images are shown in (**I**) and quantification are shown in (**J**). Data are presented as mean ± SD; n=3.

**K-L**. Neutral lipid levels were measured by Nile Red in FASN-knockdown H1299 cells examined by microscope. The representative fluorescent images are shown in (**K**) and quantification are shown in (**L**). Data are presented as mean ± SD; n=3.

*P < 0.05; **P < 0.01; ***P < 0.001; ****P < 0.0001; two-tailed t-test.

**Figure S7. FASN promotes NSCLC migration, invasion and lipogenesis in NSCLC**

**A**. Cell invasion determined by transwell assay in H157 cells treated with shRNAs against FASN or control for 24 h. Scale bars = 50 μm.

**B**. Quantification of invasion ability of FASN-knockdown H157 cells in (**A**) was analyzed by Image J. Data are presented as mean ± SD; n=3.

**C**. Cell migration determined by wound healing assay in H157 cells treated with shRNAs against FASN or control for 24 h. Scale bars = 50 μm.

**D**. Quantification of migration ability of FASN-knockdown H157 cells in (**C**) was analyzed by Image J. Data are presented as mean ± SD; n=3.

**E**. Total triglycerides assay kit showing total triglycerides level was decreased in FASN-knockdown H157 cells compared with control cells. Data are presented as mean ± SD; n=3.

**F**. Total cholesterol assay kit showing total cholesterol level was decreased in FASN-knockdown H157 cells compared with control cells. Data are presented as mean ± SD; n=3.

**G-H**. Neutral lipid levels were analyzed by flow cytometry in FASN-knockdown H157 cells treated with Nile Red. Data are presented as mean ± SD; n=3.

**I-J**. Neutral lipid levels were detected by BODIPY 493/503 probe in FASN-knockdown H157 cells examined by microscope. The representative fluorescent images are shown in (**I**) and quantification are shown in (**J**). Data are presented as mean ± SD; n=3.

**K-L**. Neutral lipid levels were measured by Nile Red staining in FASN-knockdown H157 cells examined by microscope. The representative fluorescent images are shown in (**K**) and quantification are shown in (**L**). Data are presented as mean ± SD; n=3.

*P < 0.05; **P < 0.01; ***P < 0.001; ****P < 0.0001; two-tailed t-test.

**Figure S8. IGF2BP3-FASN axis promotes NSCLC proliferation and colonization in NSCLC**

**A-B**. Cell Proliferation was evaluated by CCK8 assay in IGF2BP3-knockdown and FASN-overexpression H1299 and H157 cells for 0, 24, 48, 72, 96 and 120 h. Data are presented as mean ± SD; n=3.

**C-D**. Cell proliferation was evaluated by cell counting assay in IGF2BP3-knockdown and FASN-overexpression H1299 and H157 cells for 0, 24, 48, 72, 96 and 120 h. Data are presented as mean ± SD; n=3.

**E-G**. Colony survival was detected by colony formation assay in IGF2BP3-knockdown and FASN-overexpression H1299 and H157 cells for 10 days. The representative images are shown in (**E**) and quantification are shown in (**F** and **G**). Data are presented as mean ± SD; n=3.

**H-K**. Cell apoptosis was analyzed by flow cytometry in IGF2BP3-knockdown and FASN-overexpression H1299 and H157 cells. The representative images are shown in (**H** and **J**) and quantification are shown in (**I** and **K**). Data are presented as mean ± SD; n=3.

**L-M**. Neutral lipid levels were detected by BODIPY 493/503 probe in IGF2BP3-knockdown and FASN-overexpression H157 cells examined by microscope. The representative fluorescent images are shown in (**L**) and quantification are shown in (**M**). Data are presented as mean ± SD; n=3.

**N-O**. Neutral lipid levels were measured by Nile Red staining in IGF2BP3-knockdown and FASN-overexpression H157 cells examined by microscope. The representative fluorescent images are shown in (**N**) and quantification are shown in (**O**). Data are presented as mean ± SD; n=3.

**P**. The mice body weight in IGF2BP3-knockout and FASN-overexpression H1299 cells orthotopic implantation model was measured every three days. Data are presented as mean ± SD; n=6.

ns, no significant; *P < 0.05; **P < 0.01; ***P < 0.001; ****P < 0.0001; two-tailed t-test.

**Supplementary Tables**

**Table S1. Primer sequences used to construct plasmids in this study**

| **Name** | **Direction** | **Sequence (5' - 3')** |
| --- | --- | --- |
| shIGF2BP3-1 (Human) | Forward | CCGGCGGTGAATGAACTTCAGAATTCTCGAGAATTCTGAAGTTCATTCACCGTTTTTG |
|  | Reverse | AATTCAAAAACGGTGAATGAACTTCAGAATTCTCGAGAATTCTGAAGTTCATTCACCG |
| shIGF2BP3-2 (Human) | Forward | CCGGCGGTGAATGAACTTCAGAATTCTCGAGAATTCTGAAGTTCATTCACCGTTTTTG |
|  | Reverse | AATTCAAAAACGGTGAATGAACTTCAGAATTCTCGAGAATTCTGAAGTTCATTCACCG |
| sgIGF2BP3-1 (Human) | Forward | CACCGCTTACCTCCCACTGTAAATG |
|  | Reverse | AAACCATTTACAGTGGGAGGTAAGC |
| shMETTL3 (Human) | Forward | CCGGCGTCAGTATCTTGGGCAAGTTCTCGAGAACTTGCCCAAGATACTGACGTTTTTG |
|  | Reverse | AATTCAAAAACGTCAGTATCTTGGGCAAGTTCTCGAGAACTTGCCCAAGATACTGACG |
| shFASN-1 (Human) | Forward | CCGGCCTACTGGATGCGTTCTTCAACTCGAGTTGAAGAACGCATCCAGTAGGTTTTTG |
|  | Reverse | AATTCAAAAACCTACTGGATGCGTTCTTCAACTCGAGTTGAAGAACGCATCCAGTAGG |
| shFASN-2 (Human) | Forward | CCGGGCTACGACTACGGCCCTCATTCTCGAGAATGAGGGCCGTAGTCGTAGCTTTTTG |
|  | Reverse | AATTCAAAAAGCTACGACTACGGCCCTCATTCTCGAGAATGAGGGCCGTAGTCGTAGC |

**Table S2. Primer sequences (Human) for RT-qPCR used in this study**

| **Terms** | **Forward primer (5' - 3')** | **Reverse primer (5' - 3')** |
| --- | --- | --- |
| FASN | CTGCACTTCCATAGCCCCAA | ATGCTCAGGAAAGCCAGGTC |
| IGF2BP3 | TCGTGACCAGACACCTGATGAG | GGTGCTGCTTTACCTGAGTCAG |
| METTL3 | CTATCTCCTGGCACTCGCAAGA | GCTTGAACCGTGCAACCACATC |
| β-actin | CACCATTGGCAATGAGCGGTTC | AGGTCTTTGCGGATGTCCACGT |
| CA4 | CGATGAGAAGGTCGTCTGGACT | GCCTGACATTGTCCTTCATGCTC |
| REEP6 | GCCTGTATCTGCTGTTCGGCTA | AGTAGGTGAGCCACACAGTGTC |
| ADSL | CCGACTTTGCTAAGGAACGAGC | AGGTCATCTCGGACACGCTTCA |
| YWHAH | ACGACATGGCCTCCGCTATGAA | GCTAATGACCCTCCAGGAAGATC |
| LGALS1 | AGCAGCGGGAGGCTGTCTTTC | ATCCATCTGGCAGCTTGACGGT |
| KMT5A | GTGATTCCACCAATGCAGCCATC | GCTCCTTCGGACAGGGTAGAAA |
| CPT2 | GCAGATGATGGTTGAGTGCTCC | AGATGCCGCAGAGCAAACAAGTG |
| ACOT2 | ATGGAGACGCTCCATCTGGAGT | GTGATGCCCTTCAGGAAAGAGG |
| BPHL | ATCACGCAGTCCTGCTACTTCC | CCTGGAATGTCCATAGCCTCGA |
| ALDOA | GACACTCTACCAGAAGGCGGAT | GGTGGTAGTCTCGCCATTTGTC |
| MDH2 | CTGGACATCGTCAGAGCCAACA | GGATGATGGTCTTCCCAGCATG |
| NTHL1 | AACAGGCTGAGGTGGACCAAGA | CCAAGAGTCCATTGATCTCGTGC |
| GLUL | CTGCCATACCAACTTCAGCACC | ATAGGCACGGATGTGGTACTGG |
| APEX1 | CTGCTCTTGGAATGTGGATGGG | TCCAGGCAGCTCCTGAAGTTCA |
| CRAT | CCTACAGACCAACAAGGAGCCT | TGCATCTAGGCACACGGTGAAG |
| ACAA1 | GACAGGTCATCACGCTGCTCAA | CCAGGGTATTCAAAGACGGCAG |
| ERP29 | GCAGGATGAGTTCAAGCGTCTTG | GCTCCATGTTCAGCTTGTCACC |
| UROD | TGGTTGGGCTTGACTGGACAGT | CAGCATCTGCTTCACCAACTGC |
| CEL | CATGATCGCCTACTGGACCAAC | TCTCCAGGTAGCCGCTGTTTTC |
| MIF | AGAACCGCTCCTACAGCAAGCT | GGAGTTGTTCCAGCCCACATTG |
| GRHPR | GATGTCCTGACAGATACCACCG | TCTGCGTGAGTCCATAGCCACA |
| UGDH | TGTGATGGTGCCCATGCTGTTG | GTCCATCGAAGATAAAGGCTGGC |
| PTPRG | CTTGACCTTCGTGTGCCTCATC | GAAGGCACTTCACGGAAACGTC |
| PCBD1 | CCGTGATGCCATCTTCAAGCAG | ATGGTGGTCCAGTTTCTCAGCC |
| NSDHL | CAGTTTTCCACTGTGCGTCACC | ACGCCCTCAAAGATGACACTGG |
| HSD17B10 | CGTGTGGATGTAGCTGTCAACTG | ACCAGGCGGATCACATTGAAGG |
| GCDH | CTTCGGAGTTCTGCTTGCACAC | TCTCAGTGAGCATGTCTGCCAG |
| RAP1GDS1 | CCACAGTCTTCCTGCAAAGCCA | AGCAGCACTTCCTGGTCTTTGC |
| PRDX6 | CAGCTACCACTGGCAGGAACTT | GGAAGGACCATCACACTATCCC |
| ALDH9A1 | GCCTGGAGTATTATGCGGGCTT | CCGACTTCCAAGAGGCAATCTG |

**Table S3. Primer sequences (Human) for RIP-qPCR used in this study**

| **Terms** | **Forward primer (5' - 3')** | **Reverse primer (5' - 3')** |
| --- | --- | --- |
| FASN | CTAGGCACAGAGTAGCCCCT | TGATGTCAGGGTGGGTCCTA |
